# Co-existence of intact stemness and priming of neural differentiation programs in mES cells lacking Trim71

**DOI:** 10.1038/srep11126

**Published:** 2015-06-09

**Authors:** Sibylle Mitschka, Thomas Ulas, Tobias Goller, Karin Schneider, Angela Egert, Jérôme Mertens, Oliver Brüstle, Hubert Schorle, Marc Beyer, Kathrin Klee, Jia Xue, Patrick Günther, Kevin Bassler, Joachim L. Schultze, Waldemar Kolanus

**Affiliations:** 1 Molecular Immunology and Cell Biology, Life and Medical Sciences Institute, University of Bonn, Bonn,Germany; 2 Genomics and Immunoregulation, Life and Medical Sciences Institute, University of Bonn, Bonn, Germany; 3 Department of Developmental Pathology, Institute of Pathology, University of Bonn Medical School, Bonn, Germany; 4 Institute for Reconstructive Neurobiology, Life and Brain Center, University of Bonn and Hertie Foundation, Bonn, Germany

## Abstract

Regulatory networks for differentiation and pluripotency in embryonic stem (ES) cells have long been suggested to be mutually exclusive. However, with the identification of many new components of these networks ranging from epigenetic, transcriptional, and translational to even post-translational mechanisms, the cellular states of pluripotency and early differentiation might not be strictly bi-modal, but differentiating stem cells appear to go through phases of simultaneous expression of stemness and differentiation genes. Translational regulators such as RNA binding proteins (RBPs) and micro RNAs (miRNAs) might be prime candidates for guiding a cell from pluripotency to differentiation. Using Trim71, one of two members of the Tripartite motif (Trim) protein family with RNA binding activity expressed in murine ES cells, we demonstrate that Trim71 is not involved in regulatory networks of pluripotency but regulates neural differentiation. Loss of Trim71 in mES cells leaves stemness and self-maintenance of these cells intact, but many genes required for neural development are up-regulated at the same time. Concordantly, Trim71^−/−^ mES show increased neural marker expression following treatment with retinoic acid. Our findings strongly suggest that Trim71 keeps priming steps of differentiation in check, which do not pre-require a loss of the pluripotency network in ES cells.

In recent years, many molecular mechanisms underlying important cell fate decisions such as differentiation of embryonic stem (ES) cells have been elucidated^1^. During developmental processes including ES cell differentiation, a major model of action that has been put forward is cross-inhibitory regulation between transcription factors (TFs), which are believed to result in cell states of mutually exclusive and binary cell specifications. In such models, the induction and cooperative execution of additional TFs is required for further cell differentiation with high fidelity and specificity^2^,^3^.

However, there is also increasing evidence that such regulation is more complex in higher vertebrates including whole networks of transcriptional regulators to allow changes from one cell state to another^4^,^5^,^6^,^7^,^8^,^9^. For example, chromation immunoprecipitation DNA sequencing (ChIP-seq) of multiple TFs, in addition to well-known regulators of self-renewal (e.g. Nanog, Oct4, Sox2), revealed that TFs including Tcfcp2l1, Stat3^6^, Dax1, and Klf4^4^, are important members of a larger network of regulators securing pluripotency or maintenance of the undifferentiated state in murine embryonic stem (mES) cells. Very recently, an essential transcription factor program for pluripotency was defined by a computational approach to contain at least 12 components^10^, whereas protein-protein interaction network analysis suggested a set of 35 proteins required to keep mES cells in an undifferentiated state^11^. Clearly, a certain hierarchy among the members of these networks was observed: whereas knock-down of Dax1 and Sall4 lead to a loss of pluripotency, as assessed by loss of Oct4 and derepression of certain lineage markers, loss of Nac1 or Zfp281 did not alter the expression of the stem-cell markers Nanog and Oct4. Yet, de-repression of markers for primitive endoderm (Gata6/4), mesoderm/visceral endoderm (Bmp2) and neuroectoderm (Isl1) was observed^11^.

These findings suggested that the switch from pluripotency to early-differentiated cells is not following mutually exclusive and binary cell specification states but may rather be described as phases of overlapping programs with several checkpoints that need to be overcome to initiate final differentiation of mES cells. While TFs certainly play a major role during these processes^4^,^12^,^13^,^14^ it has become similarly clear that many other classes of regulators including chromatin proteins and regulators, DNA binding proteins^15^,^16^,^17^,^18^,^19^, miRNAs^5^,^20^,^21^,^22^,^23^ and other non-coding RNA species^24^,^25^,^26^, but also RNA-binding proteins (RBPs)^27^,^28^,^29^,^30^ are involved in such processes. In fact, when monitoring loss of Nanog over time, it became apparent that only half of the genes changed upon loss of Nanog are regulated by chromatin modification and transcription, while the remaining genes appear to be regulated by post-transcriptional, translational and post-translational regulation^31^,^28^.

An additional layer of post-transcriptional regulation within these regulatory networks is represented by ES-associated miRNAs^5^,^20^,^21^,^22^,^23^. The major ES-associated TFs Nanog, Oct4, Sox2, and Tcf3 occupy promoters of those miRNAs that are uniquely or preferentially expressed in ES cells, in particular the miRNAs of the miR290-295 cluster. In addition, miRNA-deficient ES cells display an impaired self-renewal phenotype^20^,^21^,^22^,^23^. Therefore, miRNAs contribute posttranscriptionally to the regulatory network maintaining an undifferentiated ES cell state. Overall these findings suggest a much larger regulatory network involving epigenetic^16^,^32^,^33^,^34^, transcriptional^4^,^12^,^13^,^35^,^36^, post-transcriptional and translational^37^,^38^ mechanisms of cell fate decisions in mES cells.

Very recently, the existence of different states of mES cells and a temporal overlap of pluripotency networks and early differentiation networks at the transition from stemness to differentiation have been observed both on population- and single cell-level^31^,^39^,^40^,^41^. Intermittent loss of Nanog resulted in the co-expression of genes associated with early differentiation, yet pluripotency-related gene networks were still intact^31^. Pluripotency and differentiation state fluctuations might also be modulated by miRNAs and RBPs at the post-transcriptional or translational level. However this has not been demonstrated so far.

Recently, the repertoire of RBPs in mES cells has been mapped^30^. While more than 40 members of the Tripartite motif (Trim) protein family are expressed in mES cells, only Trim25 and Trim71 were found to be RBPs^30^ . Trim71 was initially identified as a bona fide target of the miRNA let-7, which is abundantly expressed in most differentiated cell types. The suppression of Trim71 by let-7 is highly conserved and intensively studied^42^,^43^,^44^. However, very little is known about the role of the Trim71 protein in stemness regulation and during early differentiation steps.

In this study, we assessed whether the stem cell-associated RBP Trim71 is involved in processes guiding either mES cell differentiation or pluripotency. Complete deficiency of Trim71 itself did not result in phenotypic changes of mES cells and did accordingly not alter hallmarks of pluripotency such as expression of Nanog and Oct4. Global assessment of the transcriptome revealed a split phenotype in which the large ES-specific network is unaltered, while at the same time genes involved in early as well as late neural developmental steps were de-repressed. This was similarly true for the miRNA network in Trim71^−/−^ mES cells, which displayed few changes of ES-associated miRNAs, while differentiation-associated miRNAs were already induced, suggesting that loss of Trim71 resulted in a primed state towards differentiation. The functional significance of these findings is further supported by the observation that Trim71^−/−^ mES cells show indeed an enhanced differentiation towards early neural precursors induced by retinoic acid treatment *in vitro*.

We therefore conclude that the RBP Trim71 plays an important role in a multi-step process, which enables priming of differentiation processes in mES cells, whereas transcriptional and miRNA-controlled proliferation and stemness circuitries are kept intact.

## Results

### Trim71 expression and function is involved in proper development of the dorsal neural tube

**Figure 1:**
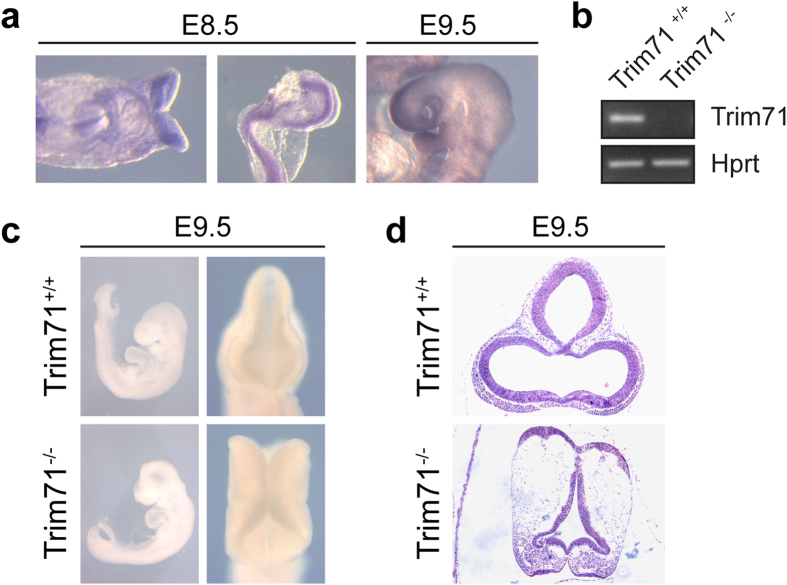
Characterization of a new Trim71 targeting allele. (**a**) *In situ* hybridization staining of wild-type embryos for endogenous Trim71 mRNA expression at E8.5 and E9.5 showing signals in the neural folds and anterior head region (30 x magnification). (**b**) Semi-quantitative RT-PCR of Trim71 mRNA and Hprt expression in wild-type versus mutant embryos at stage E9.5. (**c**) Trim71 deficient embryos exhibit a severe neural tube closure defect characterized by an open cranial (anterior) neural tube from forebrain to hindbrain (failure of closure point 2) and absence of neural tube bending at expense of dorsal elongation of the neuroepithelium. Left panel shows lateral view on whole embryos, right panel is a top view on head folds (18 and 30 x magnification, respectively). (**d**) Coronal section of the neural tube of wild-type and Trim71 deficient embryos at stage E9.5 stained with H/E (30 x magnification).

Trim71 was reported to be abundantly expressed in mES cells and during early embryogenesis, especially in the head and central nervous system region (Fig. 1a) suggesting an important function in these tissues^30^,^43^,^45^. In order to characterize the role of Trim71 in development and differentiation, we generated a novel Trim71 mouse line (Trim71^fl/fl^), which allows for conditional mutagenesis. In our gene targeting approach, the last of the four exons of the Trim71 gene was flanked by uni-directional loxP sites, enabling subsequent deletion of this exon by Cre recombination (Fig. 1b, Suppl. Fig. 1a). An *ab initio* loss-of-function mutation of the Trim71 locus was generated by mating these animals with the global deleter line *PGK-Cre*^46^. Genotype statistics of heterozygous intercrosses revealed no viable homozygous *Trim71*^*−/−*^*;PGK-Cre* offspring, indicating a 100% lethality rate of homozygously targeted embryos (Suppl. Fig. 1b). Most strikingly, starting from E9.5 all Trim71^−/−^ embryos exhibit a severe neural tube closure defect characterized by an open cranial neural tube extending from forebrain to hindbrain (cranioschisis) (Fig. 1c,d). At later stages Trim71 deficient animals show a general growth retardation of the trunk (Suppl. Fig. 1c) and we never found viable animals after E14.5 (Suppl. Fig. 1b). These phenotypes confirm and extend the ones reported earlier for non-conditional Trim71 mutant mouse lines^43^,^45^.

### Trim71 does not change major hallmarks of mES cells

**Figure 2:**
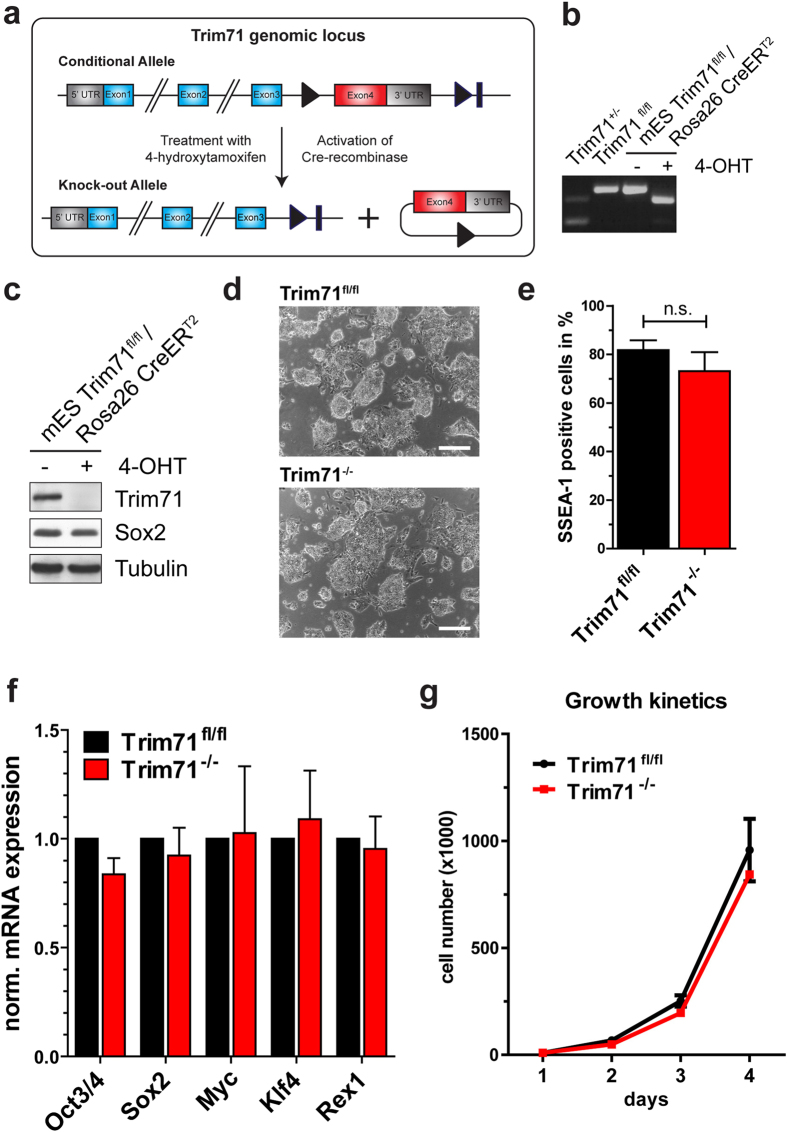
Trim71 deficient mES cells exhibit no abnormalities in the maintenance of stemness in steady state culture. (**a**) Trim71 targeting strategy for conditional deletion of Exon4 in Trim71^fl/fl^;Rosa26-CreER^T2^ mES cells. (**b**) The conversion of the Trim71 allele can be efficiently induced *in vitro* by the application of 250 nM 4-Hydroxytamoxifen for 48 hours as shown by genotyping PCR. Upper band: floxed allele; middle band: knock-out allele; lower band: wild-type allele. (**c**) As a consequence, Trim71 protein cannot be detected after 4-OHT treatment. The stem cell marker Sox2 is steadily expressed in both conditions. (**d**) Bright field images of Trim71^fl/fl^ and Trim71^−/−^ mES cells on gelatin in MEF conditioned medium (bars represent 200 μm). **(e)** SSEA-1 positive cells measured by flow cytometry. Data represent mean + SEM (n = 5); unpaired Student’s t-test; n.s. not significant. (**f**) RT-qPCR analysis of the stemness markers Oct4, Sox2, c-Myc, Klf4, Rex1. Gapdh was used as housekeeping gene and data are normalized to control cell expression. Data represent mean + SEM (n = 3). **(g)** Examination of growth kinetics of Trim71^fl/fl^ versus Trim71^−/−^ mES cells by cell counting (n = 3 independent experiments).

To assess the role of Trim71 in stem cells, we derived conditional Trim71 mES cell lines by crossing Trim71^fl/fl^ with Rosa26-CreER^T2^ animals. The inner cell masses of blastocysts were used to derive mES cell lines. All experiments described in the following were performed with 2-5 individual clones. For the induction of Cre-mediated recombination, cells were treated with 250 nM 4-Hydroxytamoxifen (4-OHT), or with DMSO as a solvent control for 48 hours, respectively (Fig. 2a). The ablation of Trim71 was confirmed by genotyping and the observed recombination rates were reliably higher than 90% in 4-OHT treated mES cells (Fig. 2b). Accordingly, Trim71 protein was not detectable in 4-OHT treated samples using western blot analysis (Fig. 2c), whereas Sox2 expression remained unaltered.

Surprisingly, Trim71 deficient mES cells displayed no morphological differences in comparison to control cells and formed typical mES cell colonies (Fig. 2d). When analyzing the surface expression of the pluripotency marker stage-specific embryonic antigen 1 (SSEA-1) by flow cytometry we could not detect any differences between Trim71^fl/fl^ and Trim71^−/−^ mES cells further indicating that Trim71 did not change the stem cell phenotype (Fig. 2e). Next we also measured the expression of well-established pluripotency-associated factors such as Oct4, Sox2, c-Myc, Klf4, and Rex1 by RT-qPCR^47^. However, we did not observe a differential expression of these stemness markers in Trim71^−/−^ cells in comparison to Trim71^fl/fl^ mES cells (Fig. 2f). Recently it was shown that siRNA-mediated Trim71 knock-down in mES cells resulted in decreased proliferation mainly mediated by induction of the inhibitory cell cycle regulator Cdkn1a (encoding for p21)^48^. When assessing the proliferation in Trim71^−/−^ and Trim71^fl/fl^ mES cells, we again did not detect any defects in Trim71 deficient cells (Fig. 2g). Overall, loss of Trim71 in mES cells does not result in phenotypical impairments with respect to stem cell characteristics.

### Loss of Trim71 results in transcriptional deregulation of mES cells

**Figure 3:**
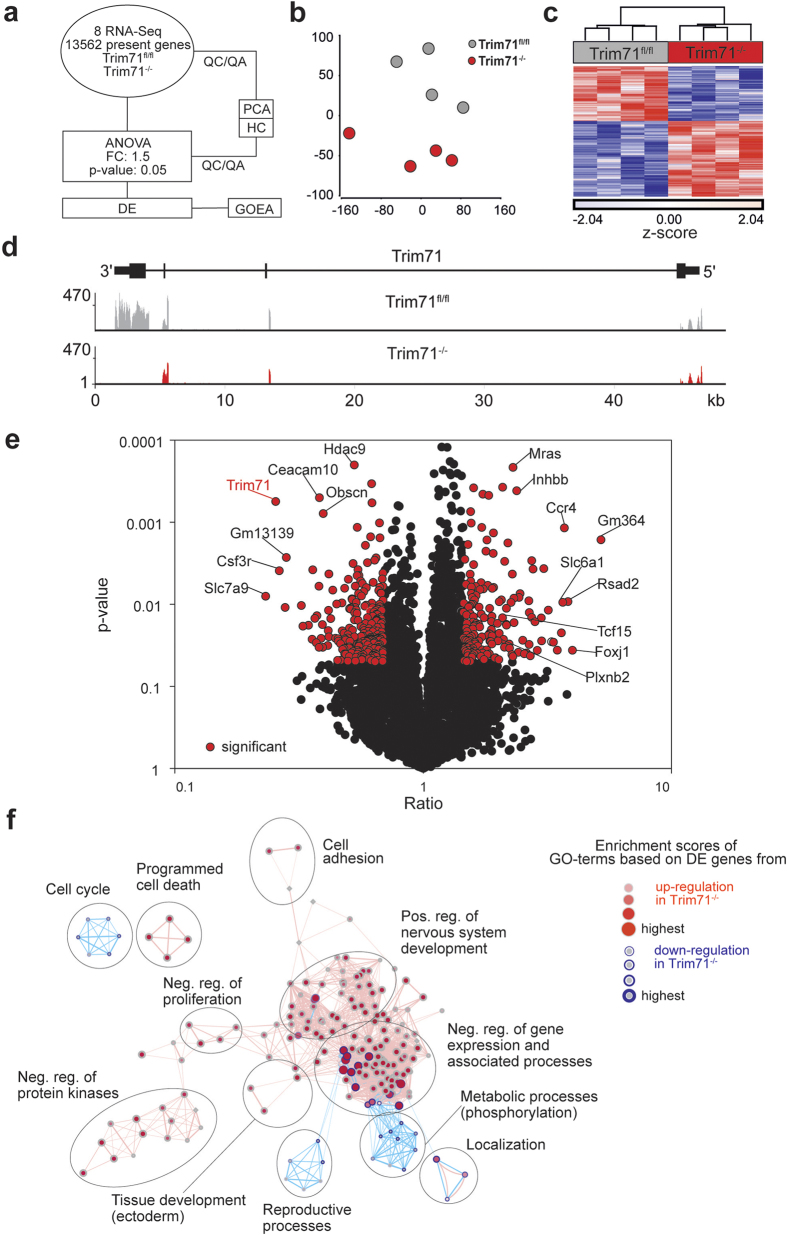
Transcriptome analysis of Trim71^−/−^ ES cells in comparison to wild-type ES cells. (**a**) Schema describing the workflow for Fig. 3. (**b**) Genomic distribution of reads across the Trim71 gene locus in the two ES cell types. (**c**) Principal component analysis (PCA) based on present and variable genes. (**d**) Hierarchical clustering (HC) showing the z-transformed expression values of the top 1.000 present and variable genes, colored from blue to red. (**e**) Volcano plot of all present genes. Genes significantly up- or downregulated (fold change |FC| > 1.5; p-value < 0.05) in Trim71^−/−^ over Trim71^fl/fl^ ES cells are marked in red or blue. Gene symbols of the most significantly regulated genes are added to the plot. (**f**) Network visualization of Gene Ontology Enrichment Analysis (GOEA) based on 166 most differentially upregulated and 216 most downregulated genes using BiNGO and EnrichmentMap. Enriched GO-terms based on upregulated genes are depicted by red nodes, enriched GO-terms on downregulated genes as blue nodes where color and size represent the corresponding FDR-adjusted enrichment p-value (q-value). Overlap of genes between nodes is indicated by edge thickness. Abbreviations: QC quality control, QA quality assurance, DE differential expression.

Our analysis so far suggested that loss of Trim71 does not change stemness in mES cells. To address this on a global level, we generated genome-wide transcriptome data by quantitative RNA-sequencing in Trim71^fl/fl^ and Trim71^−/−^ mES cells and performed an extended bioinformatic analysis as outlined in Fig. 3a. 13.558 transcripts were identified to be present in either Trim71^fl/fl^ or Trim71^−/−^ mES cells. We first verified for Trim71^−/−^ mES cells at the gene locus level that exon 4 is indeed deleted, resulting in a loss of transcripts of this exon, while the other exons are still transcribed (Fig. 3b). These results also explained residual expression of Trim71 when applying gene-centered models for expression analysis (Suppl. Table 1). Unbiased principle component analysis (PCA) based on 13.558 transcripts (Fig. 3c) and hierarchical clustering on the 1.000 most variable (p < 0.05) present genes (Fig. 3d) clearly revealed significant transcriptional changes in Trim71 deficient cells. Using a one-way ANOVA-model we determined differentially expressed genes between the two mES cell types and plotted the ratios against the p-values in a volcano plot (Fig. 3e). Although Trim71 was previously described only as a repressor of mRNA stability and function, we found that the number of genes significantly up- or downregulated with a fold change of ≥1.5 was comparable (166 and 216 genes, respectively). As expected, Trim71 was among the most significantly reduced genes in Trim71^−/−^ mES cells. Among the most elevated genes was Foxj1, a TF that has not been implicated in stem cell biology, but rather in embryonic and postnatal neurogenesis^49^, suggesting that Trim71 serves to control neural differentiation in mES cells. To understand better which biological processes might be deregulated by the loss of Trim71 on a global scale, we performed Gene Ontology Enrichment Analysis (GOEA) followed by network visualization (Fig. 3f). Indeed, one of the large clusters of GO-terms was associated with positive regulation of morphogenesis involved in nervous system development and another smaller cluster with ectoderm development again supporting the notion that loss of Trim71 might lower the threshold towards induction of neurogenesis in mES cells. Additionally, loss of Trim71 leads to a downregulation of genes implicated in reproductive processes.

### The core mES cell network is not affected by the loss of Trim71

**Figure 4:**
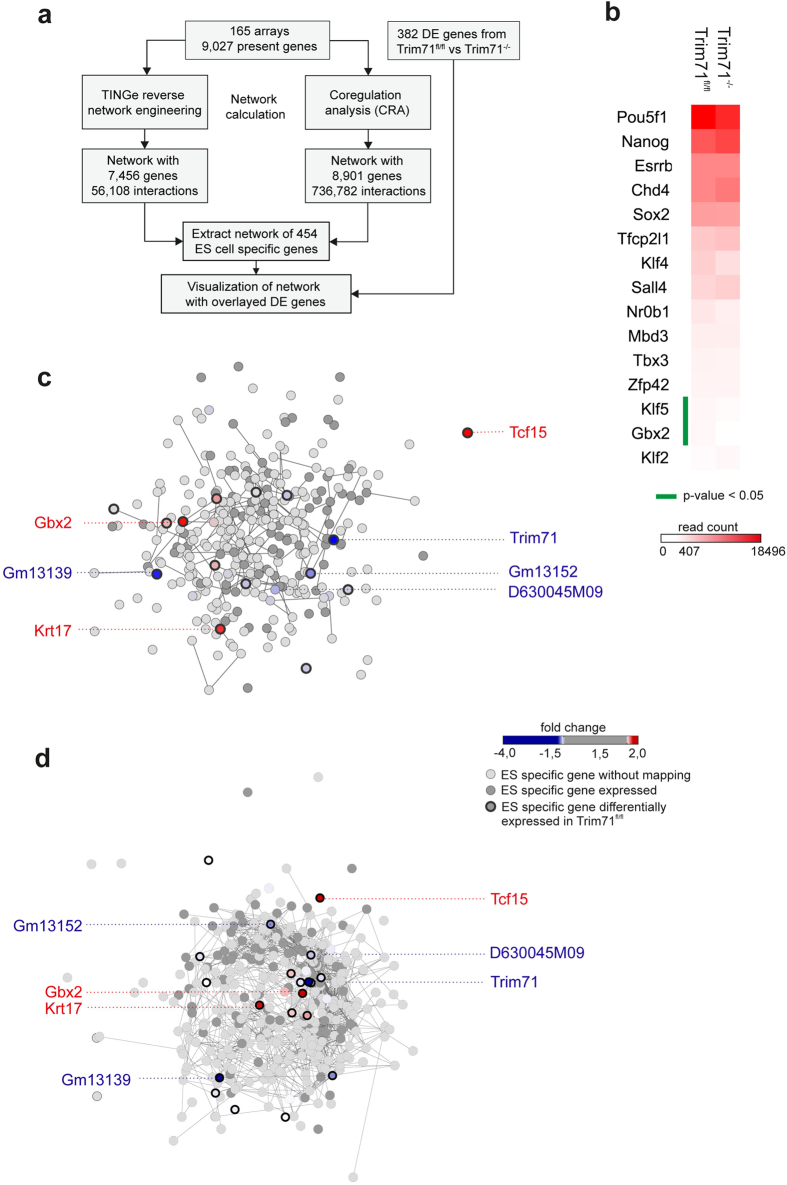
Network overlay of embryonic development with differentially expressed genes in Trim71^−/−^ ES cells compared to Trim71^fl/fl^ mES cells. (**a**) Schema describing the workflow for Fig. 4. (**b**) Heatmap of absolute mRNA expression values of important transcriptional regulators of mES cells. (**c**) Reverse engineered network based on the TINGe algorithm was generated using a previously published dataset. The network topology of 454 mES cells-specific genes was visualized using Cytoscape. Light grey nodes represent nodes were data from the Trim71^fl/fl^ and Trim71^−/−^ mES cells dataset could be mapped. Genes differentially expressed between Trim71^fl/fl^ and Trim71^−/−^ mES cells are color^-^coded in red (upregulated in Trim71^−/−^ mES cells) respectively in blue (downregulated). (**d**) Using the same dataset, a co-regulation network was calculated and the topology of 454 mES cells-specific genes visualized using Cytoscape. Color-coding of nodes is identical to Fig. 4c.

The large number of differentially expressed genes in Trim71^−/−^ mES cells (Fig. 3) despite any evidence for changes in stemness (Fig. 2) was rather surprising. To further address the impact of Trim71 on stemness (Fig. 4a) we made use of a recently suggested model of stemness^10^ and previous datasets describing mES cell biology^50^. We visualized the mean expression values in Trim71^fl/fl^ and Trim71^−/−^ mES cells as a heatmap for a set of 15 transcriptional regulators that were defined as the essential transcription factor program for stemness (Fig. 4b)^10^. Except for expression changes of the transcription factors (TFs) Klf5 and Gbx2, loss of Trim71 did not change expression of these major mES cell-related TFs, further supporting that loss of Trim71 does not result in loss of the mES cell phenotype. To better understand this on a larger scale we next used existing datasets (165 microarray expression profiles from two previously published datasets, GSE19836 and GSE32015)^51^ to generate mES cells specific gene networks and mapped transcriptional changes between Trim71^fl/fl^ and Trim71^−/−^ mES cells onto these networks (Fig. 4a). As a first approach we performed reverse network engineering (RNE) utilizing TINGe (Tool for Inferring Network of Genes)^52^. We used the 9.027 genes present in at least one sample (Suppl. Table 2) to generate a so-called all-versus-all network (p-value 10^−6^), by predicting interactions based on mutual information between each gene pair computed from the expression profiles (Suppl. Fig. 2; for further technical details see Suppl. Methods). We identified 56,108 interactions resulting in an average degree of connectivity of 15,050, meaning one gene is involved in about 16 transcriptional interactions on average using a mutual information cut-off of 0.4 (Suppl. Fig. 2b). Since the entire network is too complex to be visualized, we summarized the network statistical properties (Suppl. Fig. 2). Next, we reduced the network to a set of 454 unique genes that were previously defined by De Cegli *et al*.^51^ to be ES-cell specific and visualized these genes including only direct interactions between genes within the set of genes (Fig. 4c). For 243 of these genes (54%) showing significant expression (over background) in our RNA-seq data, we could map fold-change differences in expression between Trim71^fl/fl^ and Trim71^−/−^ mES cells onto the network (Fig. 4c, Suppl. Table 3). In this central mES cell-specific network we only found 4 genes including Trim71 itself to be regulated with a FC < −1.9. Tcf15, Gbx2 and Krt17 were the only genes found upregulated with a FC > 1.9. Tcf15 was recently shown to prime pluripotent cells for differentiation^53^,^54^, and Gbx2 is a well-known marker for both, undifferentiated mES cells as well as for neural development^55^,^56^,^57^. We validated these finding with a second approach by building a co-regulation network based on a Markov clustering algorithm^58^. This model resulted in a very dense network of 8,901 genes with 736,782 interactions of which we extracted the 454 mES cell-specific genes (nodes) with their direct interactions within this set of genes (Fig. 4d). We also mapped the few differentially expressed genes between Trim71^fl/fl^ and Trim71^−/−^ mES cells onto this network. Both, the co-regulation model and the reverse engineering approach suggested similar distances between genes differentially expressed in Trim71^−/−^ mES cells (Fig. 4c,d). Together, integration of our data with prior mES cell-associated datasets and models clearly support that a loss of Trim71 does not alter the majority of genes of the central gene network of mES cells.

### Integrative network analysis of neuronal cell development supports a primed state of Trim71^−/−^ mES cells

**Figure 5:**
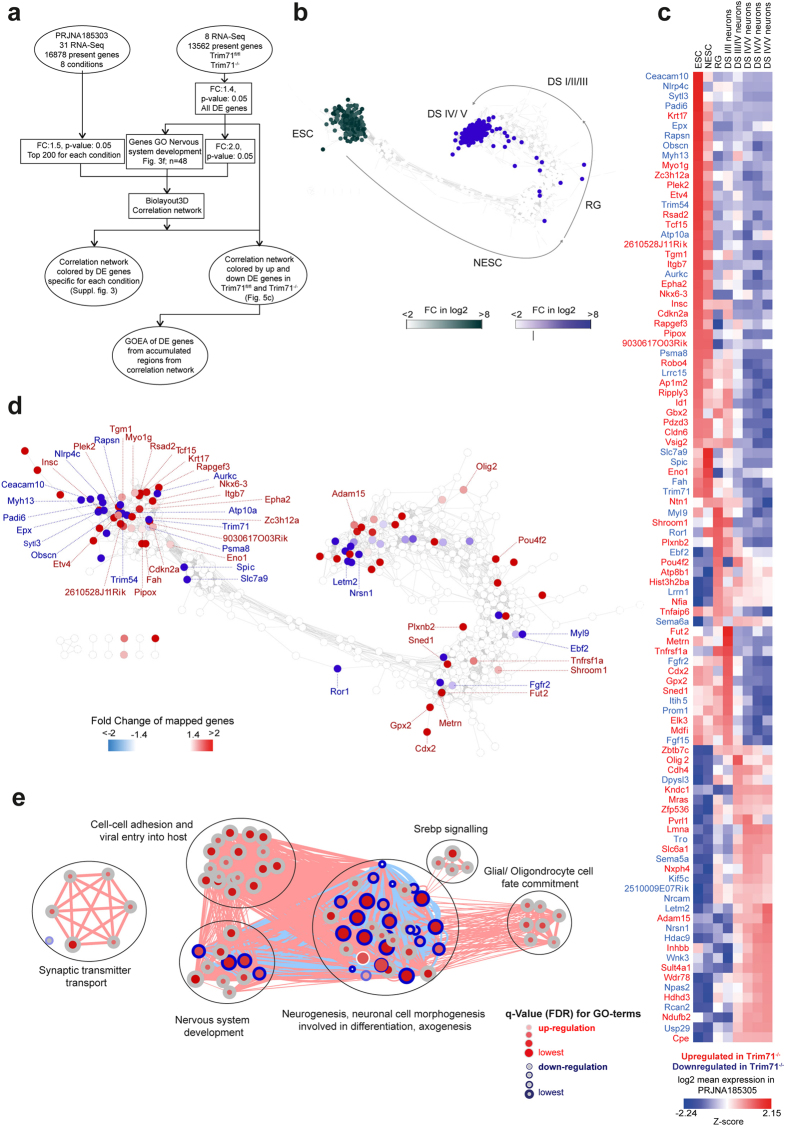
Network overlay of neuronal cell development with differentially expressed genes between Trim71^−/−^ and Trim71^fl/fl^ mES cells. (**a**) Schema describing the workflow for Fig. 5. A co-regulation network was generated based on a previous dataset covering the process of neuronal development from mES cells^63^. The resulting network was visualized in Cytoscape. (**b**) Overlay of the genes specific for the mES cells stage (green) or mature developmental stage IV/V neurons (blue). Arrows indicate the progression of genes from early to later stages in neuronal development. (**c**) Correlation network from (b) overlaid with up and down regulated genes in Trim71^−/−^ ES cells (|FC| > 1.4, p-value < 0.05). (**d**) Gene expression values during neuronal development of significantly regulated genes in Trim71^−/−^ mES cells were visualized as a heatmap. Gene names for genes upregulated in Trim71^−/−^ mES cells are colored in red, those downregulated in blue. (**e**) Network visualization of Gene Ontology Enrichment Analysis based on differentially expressed genes in Trim71^−/−^ ES cells associated with late-stage neuronal development in (c) using BiNGO and EnrichmentMap. Enriched GO-terms are depicted by red nodes, where color and size represent the corresponding FDR-adjusted enrichment p-value (q-value). Overlap of genes between nodes is indicated by edge thickness.

Next we compiled two additional datasets (GSE11523, GSE58300) that allowed us to compare changes during germ layer differentiation towards endoderm, ectoderm or mesoderm (Suppl. Fig. 4a). Intersecting differential gene expression between ES cells and the three germ layers with differentially expressed genes between Trim71^−/−^ and Trim71^fl/fl^ mES cells revealed candidate genes for all three comparisons (Suppl. Fig. 4b). GOEA of these genes showed significant enrichment of general GO terms associated with developmental processes in all three germ layers (*e.g*. ‘developmental process’). However, the highest enrichment scores were obtained for the ectoderm and only in the comparison with the ectoderm data, we identified more specific terms such as ‘CNS development’, ‘regulation of CNS development’ or ‘regulation of branching morphogenesis of a nerve’ (Suppl. Fig. 4c-e). Furthermore, we found the highest overlap between the data obtained from the PRJNA185305 data set (Fig. 5) with the data derived from the ES cell to ectoderm differentiation (Suppl. Fig. 4d).

After establishing that loss of Trim71 did not alter the central stem cell network (Fig. 4) we next addressed whether Trim71^−/−^ mES cells are geared towards neural development as suggested by GOEA (Fig. 3f). For this purpose, we utilized a recently published dataset of longitudinal RNA sequencing of transcriptional regulation during neurogenesis starting from mES cells (Fig. 5a)^59^. This study includes 8 defined time points from mES cells via neuroepithelial stem cells, developmental stage (DS) I/II neurons to maturing DS IV/V neurons. The generation of a co-regulation network based on the top 200 differentially expressed genes of each time point allowed the topological visualization of transcriptional changes during this developmental process (Fig. 5b). In order to understand the network topology better we mapped fold change differences in expression between the different time points revealing developmental stage specific sub-networks (Fig. 5b, Suppl. Figure 3). Genes associated with mES cells clustered together in a large sub network (Fig. 5b; dark green nodes), while *e.g*. genes expressed in maturing DC IV/V neurons formed the most distant sub network (Fig. 5b; blue nodes), whereas genes expressed at time points in-between were allocated to smaller clusters in between (Suppl. Fig. 3). The definition of the network topology allowed us to map fold changes of the corresponding genes between Trim71^fl/fl^ and Trim71^−/−^ mES cells to particular stages in neuronal development (Fig. 5c). Utilizing the genes differentially expressed in Trim71^−/−^ mES cells that mapped onto the network, we also generated a heatmap of gene expression in the original neurogenesis dataset (Fig. 5d). A large number of genes elevated in Trim71^−/−^ mES cells including Krt17, Myo1g, Zc3h12a, Plek4, Etv4 or Rsad2 are genes mainly expressed in mES cells and neural stem (NS) cells. However, we also found genes upregulated in Trim71^−/−^ mES cells that are found to be elevated at later time points (*e.g*. Adam15, Olig2, Pou4f2) even up to the state of DS IV/V neurons. Using differentially expressed genes in Trim71^−/−^ mES cells (as bait for GOEA) mapping onto network clusters that were related to later developmental stages (Fig. 5e) resulted in 6 major clusters of GO-terms of which 5 were related to either neural development or function further supporting that loss of Trim71 did indeed prime mES cells towards neuronal development.

Taken together, these findings strongly support that the loss of Trim71 mainly initiates a neuronal development program in mES cells, albeit this program does not seem to follow the coordinated development normally seen, as highlighted by differential expression of genes throughout the neural development path.

### Trim71 response elements located in the 3’UTR lead to repression of target mRNAs

**Figure 6:**
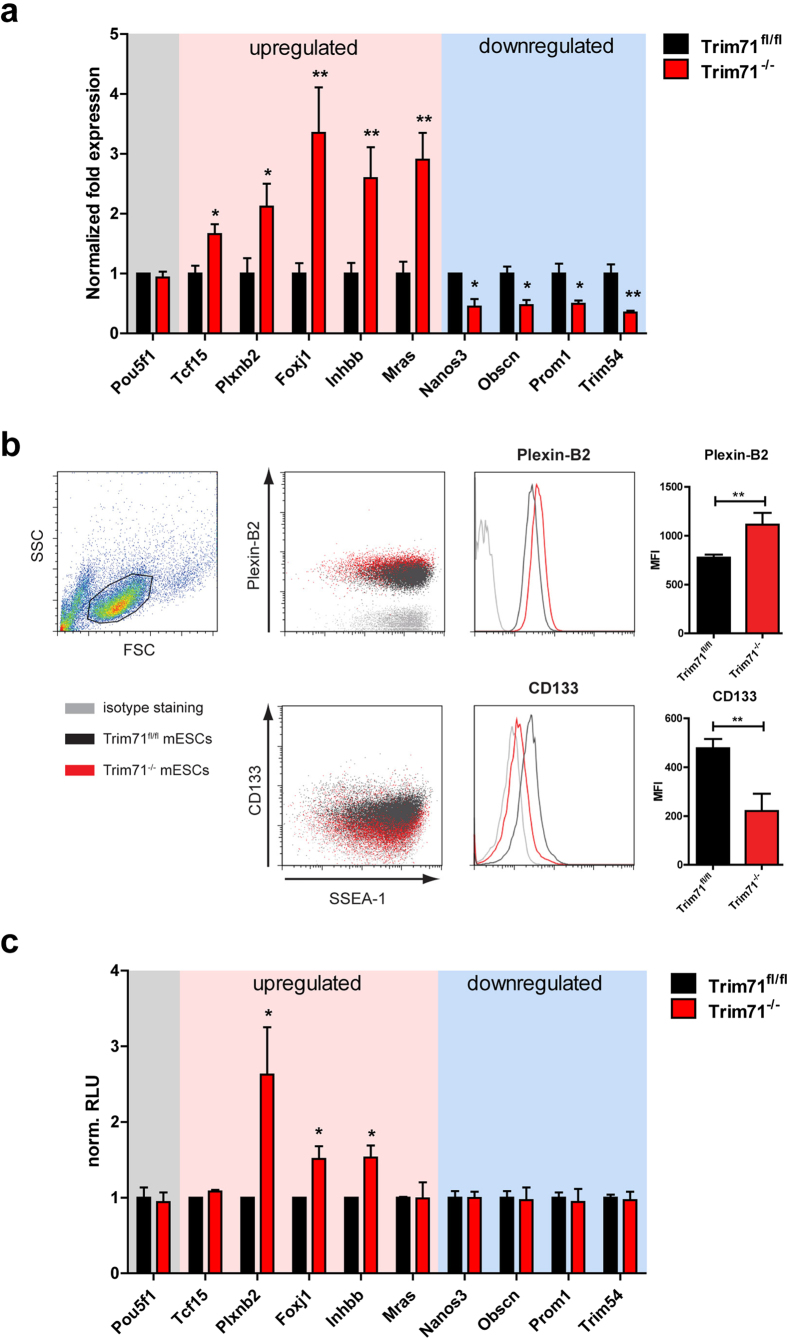
Trim71 acts on the 3’UTR of regulated mRNAs. (**a**) Expression validation of a panel of differentially expressed genes and Pou5f1 in Trim71^fl/fl^ and Trim71^−/−^ mES cells by RT-qPCR analysis. Data are mean +SEM of 3 -6 independent experiments. (**b**) Representative FACS staining of the stemness marker SSEA-1 together with CD133 or Plexin B2 respectively in Trim71 deficient and control mES cells. Graphs on the right show quantification of mean fluorescence intensity (MFI) (mean + SEM of 4 independent experiments.) (**c**) Relative light units (RLU) of different 3’UTRs using luciferase reporter constructs normalized to control cell levels (n = 3–6). For all experiments the significance was tested with the Student’s t-test with *p < 0.05, **p < 0.01, ***p < 0.001.

As mentioned earlier, the majority of differentially expressed genes are not essential for mES cell stemness but rather specific for differentiated cell states. Therefore, we aimed to investigate the characteristics of these gene regulations in more detail. We selected a group of up- and downregulated genes (Suppl. Fig. 5a) for independent expression analysis by qPCR and could in each case confirm the data obtained by RNA-seq analysis (Fig. 6a). Next, we wanted to show that the altered expression of these genes is not the result of spontaneous differentiation within the Trim71^−/−^ mES cell population but inherent to all cells. Therefore, we performed FACS staining of one up- and one downregulated gene candidate (Plexin B2 and CD133) in together with SSEA-1 staining for undifferentiated cells. We found that Plexin B2 shows indeed a higher surface expression in Trim71^−/−^ mES cells on the entire cell population resulting in a right-shift of the histogram without split signals (Fig. 6b). On the other hand, CD133, the protein coded by the Prom1 gene, shows already a very low basal expression in control cells, which is even more decreased in Trim71^−/−^ mES cells. Taken together, this illustrates that the observed expression changes are occurring in the undifferentiated mES cell population with intact stemness marker expression. Moreover, it also shows that differential gene expression observed in Trim71 deficient mES cells lead to a relevant change in protein output and might thus directly influence cell behavior.

Since Trim71 was formerly proposed to be an RBP, we next tested whether Trim71 mediates expression regulation via interaction with the 3’UTR of the mRNAs of our validated candidates. Therefore, the 3’UTRs were cloned behind a Renilla luciferase cDNA for quantification of expression in Trim71^fl/fl^ and Trim71^−/−^ mES cells (Suppl. Fig. 5b). While three out of five upregulated genes showed corresponding expression regulation in Trim71^−/−^ mES cells, this was not the case for any of the downregulated gene candidates (Fig. 6c). This indicates that Trim71 acts primarily as a repressor of mRNA stability and expression. On the other hand, upregulation of mRNA targets likely is the result of other mechanisms.

### Loss of Trim71 induces changes of the miRNA network

**Figure 7:**
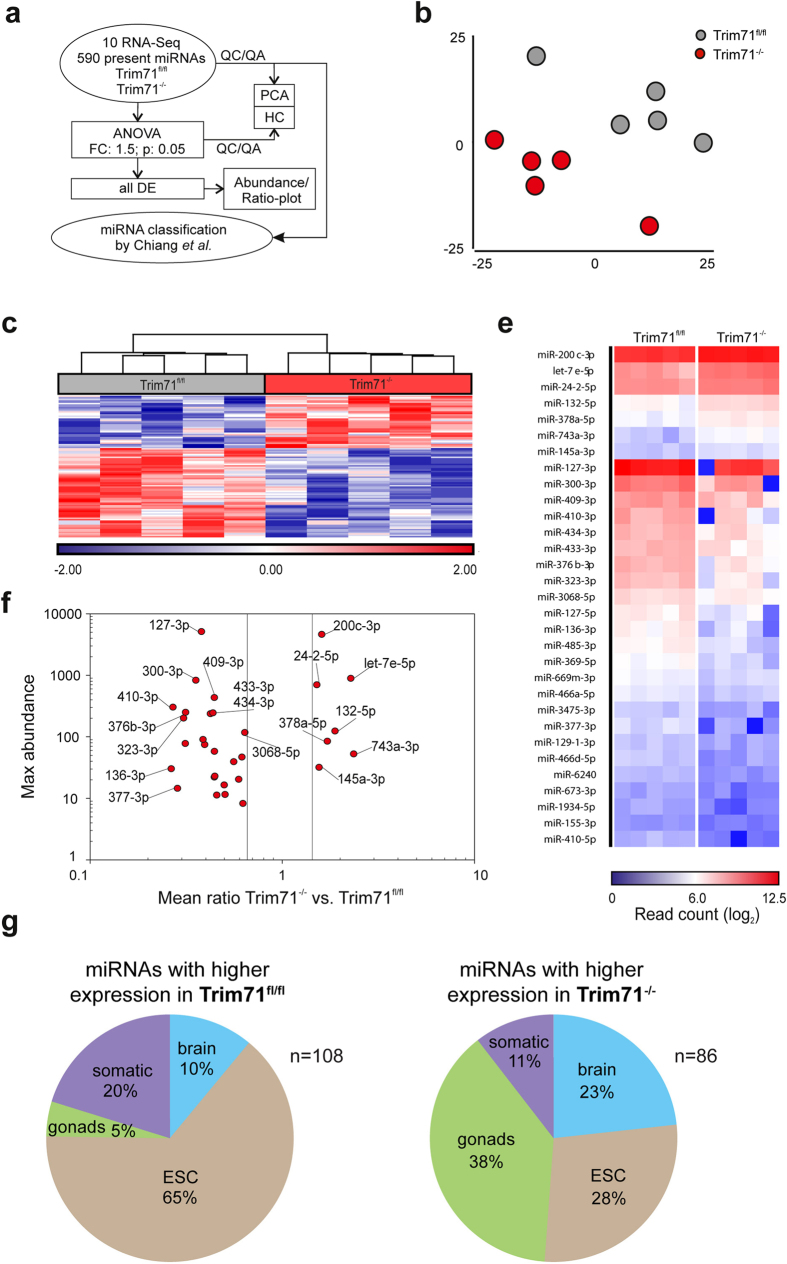
MiRNAome analysis of Trim71^−/−^ and Trim71^fl/fl^ mES cells. (**a**) Schema describing the workflow for Fig. 7. (**b**) PCA based on present and v**a**riable genes. (**c**) Hierarchical clustering of the top 100 present and most variable genes and visualization as a heatmap showing the z-transformed expression values colored from blue to red. (**d**) Visualization of log2-transformed read counts of differentially expressed miRNAs (fold change (FC) < −1.5 or > 1.5; p-value < 0.05) between Trim71^fl/fl^ and Trim71^−/−^ mES cells as a heatmap. (**e**) Abundance/ratio plots to visualize differentially expressed miRNAs (p-value <^ ^0.05) in relation to their expression levels in the respective cell types. For each miRNA the maximum abundance of the two cell types compared is plotted. (**f**) miRNAs were classified according to Chiang *et al*. into categories with miRNAs mainly expressed in gonads, brain, other somatic tissue or mES cells. miRNAs either expressed at higher levels in Trim71^fl/fl^ (n = 108, left chart) or Trim71^−/−^ (n = 86, right chart) were categorized and distribution plotted as a pie chart.

Apart from its role as an RNA binding protein, Trim71 had been implicated in the regulation of miRNA expression by acting as an E3 ubiquitin-ligase for the RISC protein Ago2. Indeed, we could confirm a co-localization of Trim71 and Ago2 in cytoplasmic p-bodies, as well as direct binding of Trim71 and Ago2 (Suppl. Fig. 6a,b). However, in line with other studies we found no alterations in Ago2 protein levels as well as of Ago2 mRNA expression after Trim71 deletion in mES cells, showing that Ago2 turnover is not regulated by Trim71 (Suppl. Fig. 6c,d)^45^,^48^,^60^. However, this does not rule out the possibility of an involvement of Trim71 in the regulation of miRNA maturation and functionality. Hence, we performed global RNA-sequencing of short RNAs and mapped the information to mirBASE to determine the genome-wide miRNAome of Trim71^fl/fl^ and Trim71^−/−^ mES cells (Fig. 7a). A total of 590 miRNAs were identified to be present (cutoff 10) in at least one sample (Suppl. Table 4). Expression values ranged between 1 and 1,397,610. PCA of all miRNAs (Fig. 7b) and hierarchical clustering of the 100 most variable miRNAs (Fig. 7c) revealed a group-related structure in the data supporting biological relevant differences in miRNA expression between Trim71^fl/fl^ and Trim71^−/−^ mES cells. To directly determine differentially expressed miRNAs (DE miRNAs) an ANOVA model was applied resulting in 7 and 24 significantly up- and downregulated miRNAs in Trim71^−/−^ mES cells, respectively (Fig. 7d). Of those miRNAs, we plotted the abundance of expression against the ratio of expression, illustrating that differential miRNA expression was not restricted to low abundance miRNAs (Fig. 7e). Among the miRNAs significantly upregulated in Trim71^−/−^ mES cells we identified a let-7 family member (let-7e) which like other let-7 members has been linked to mES cells differentiation^61^. Moreover, three additional miRNAs (mir-132, mir-24-2, mir-200) found to be upregulated in Trim71^−/−^ mES cells had already been linked to differentiation processes in ES cells or embryogenesis^62^,^63^,^64^ further supporting the notion that the loss of Trim71 poises mES cells towards differentiation.

To further investigate the functional implications of Trim71 deficiency, we categorized all miRNAs using miRNA-expression profiling from different tissues in mice^65^,^66^. As proposed by Jouneau *et al*.^65^ we merged the existing 8 different tissues into five different categories (ES, gonads, brain, other somatic tissues and unspecified) and classified miRNAs based on their highest expression in one of the five categories (Fig. 7f). Functional categories could be assigned to 442 of the 590 miRNAs present in our dataset of which 247 were annotated as ubiquitously expressed and therefore not further considered. In Trim71^fl/fl^ mES cells, the majority of mES cell-associated miRNAs (65%) were higher expressed than in Trim71^−/−^ mES cells. In contrast, miRNAs categorized as brain- and gonad-specific were expressed higher in Trim71^−/−^ mES cells, further illustrating the changes in the miRNA expression landscape. Taken together, loss of Trim71 not only changes the global transcriptional landscape but also the miRNA spectrum.

### Trim71 deficiency enhances neuroectodermal differentiation *in vitro*

**Figure 8:**
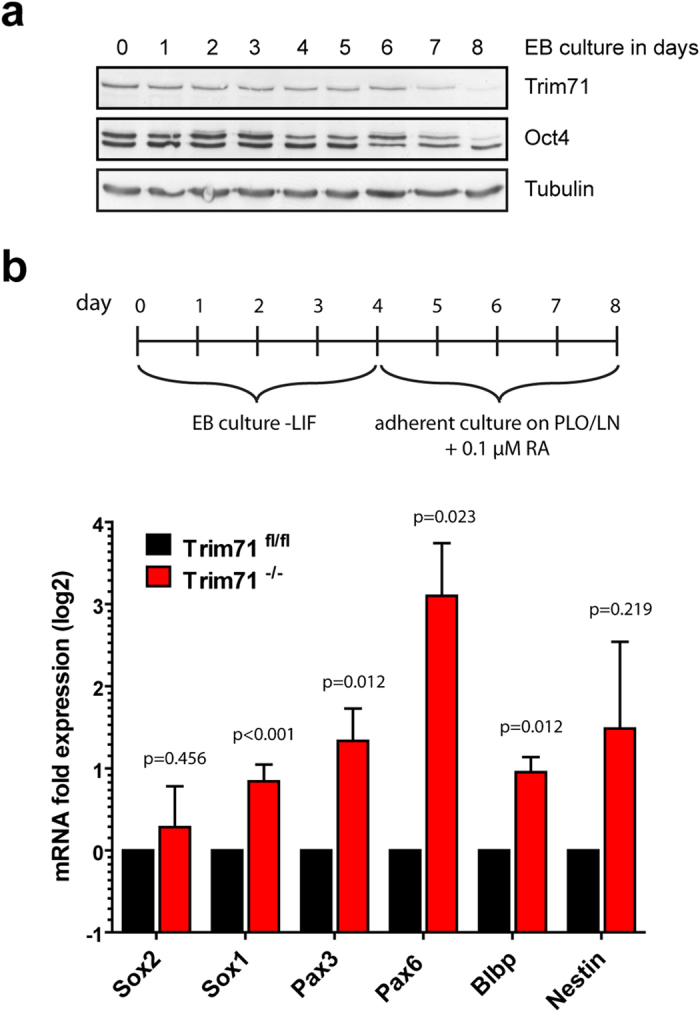
Trim71 knock-out leads to premature neuroectodermal differentiation in the mES cell model. (**a**) Trim71 is down-regulated in the course of unbiased differentiation as embryonic bodies (EBs). (**b**) Neuroectodermal differentiation was induced by 4 days EB culture with subsequent plating on PLO/LN coated dishes in the presence of RA for another 4 days results in higher expression of neuroectodermal marker genes in Trim71 deficient cells. Data are mean + SD from 5 independent experiments; p-values were calculated by Student’s t-test.

Next, we analyzed if the observed changes might have an impact on the behavior after induction of differentiation *in vitro*. Therefore, we induced unbiased differentiation by culturing mES cells as embryoid bodies (EBs) in the absence of LIF. In wild-type mES cells, differentiation leads to a downregulation of Trim71 and Oct4 in the course of several days (Fig. 8a). Considering the striking effect of Trim71 deficiency on neuroectodermal development *in vivo*, we analyzed the formation of the neuroectodermal lineage in more detail. For this purpose, the mES cells were cultured as EBs for four days and then dissociated and plated on Poly-L-ornithine/Laminin coated dishes in medium containing 0.1 μM retinoic acid (RA). Sox2 is markedly expressed in both, undifferentiated mES cells and neuroectodermally committed cells, whereas it is rapidly down-regulated during mesendodermal differentiation^67^. On the other hand, the RA stimulated differentiation induces expression of certain marker genes characteristic for early neural stem cells and radial glia cells such as the transcription factors Sox1, Pax3, and Pax6, the metabolic protein Blbp (also named Fabp7) and the intermediate filament protein Nestin^68^,^69^,^70^. We measured the mRNA expression of those genes in Trim71^fl/fl^ and Trim71^−/−^ mES cells after 8 days of differentiation and observed an increased expression of Sox1, Pax3, Pax6, Blbp and Nestin, but not of Sox2 in Trim71^−/−^ cells (Fig. 8b). These data demonstrate that Trim71 deficiency leads to enhanced expression of neuroectodermal marker genes. With an alternative differentiation approach using monolayer cultivation with N2B27 stimulation we also observed higher expression of the early neural marker genes Sox1 and Pax6 (Suppl. Fig. 7a-b).

The results shown above suggested a role of Trim71 in the regulation of early neural development starting at the pluripotent ES cell level. To assess the role of Trim71 in later stage multipotent neural precursors, primary radial glia-like neural stem cells (NS cells) were isolated at E14.5 and further cultured in the presence of EGF and FGF2 *in vitro*^71^. In contrast to mES cells, the NS cells robustly expressed let-7a (Suppl. Fig. 8a), but not the ES cell specific miRNAs miR-294 and miR-302a. Using 4-OHT we generated Trim71^−/−^ NS cells (Suppl. Fig. 8b). It was found that wild-type and Trim71^−/−^ NS cells show similar morphology (Suppl. Fig. 8c) and marker expression (Suppl. Fig. 8d). However, in agreement with a significant let-7a expression, Trim71 mRNA and protein were barely detectable in Trim71^fl/fl^ and Trim71^−/−^ NS cells. This suggests that the function of Trim71 in neural development clearly precedes the differentiation of neural precursors and its expression is consequently down-regulated at later stages of neural differentiation. Accordingly, wild-type NS cells already showed loss of Trim71 expression (Suppl. Fig. 8e).

## Discussion

Introducing the first conditional Trim71 knock-out model (Trim71^fl/fl^), we unequivocally demonstrate that complete loss of Trim71 de-represses pro-differentiation genes, yet the stem cell-associated transcription factor network and even the extended stem-cell gene expression network are not disturbed. The persistence of these important hallmarks of stemness in Trim71^−/−^ mES cells was further substantiated by an unaltered growth rate and no changes in morphology and colony formation. At the same time loss of Trim71 resulted in a significant change of the global transcriptome. In particular, we found regulation of genes involved in neurodevelopment and genes associated with reproductive processes. A similarly primed state was determined by changes in miRNA expression reflecting a shift towards miRNAs that have been previously linked to germ cell and neural development while the main mES cell miRNA cluster (miR-290–295 cluster) was not altered significantly. Let-7 family members were expressed at higher levels, and let-7e in particular was significantly elevated in Trim71^−/−^ mES cells.

Since the first phenotypic description of the Trim71 homolog lin-41 in *C. elegans*, the molecular function of Trim71 has been subject to intensive debate. However, in the last couple of years, a number of publications shed some light on this issue, since Trim71 has been implicated in a variety of different processes such as miRNA and mRNA regulation, as well as FGFR-signaling^45^,^60^,^72^. Importantly, Trim71 has also been characterized as one of the main RNA binding proteins of mES cells^30^. By establishing Trim71 knock-out mES cell lines derived from Trim71 conditional mouse embryos, we now provide a cell-autonomous model for the analysis of Trim71 function, allowing for the investigation of Trim71 dependent mechansims in a well-defined and relevant setting. In contrast to earlier suggestions, we conclude that Trim71 is not a *bona fide* pluripotency factor^48^,^60^, as Trim71 deficient mES cells show no obvious abnormalities in cell proliferation, self-renewal, morphology or the expression of important marker genes. Furthermore, we found no evidence for the regulation of Ago2 protein levels in Trim71^−/−^ mES cells, although specific subsets of miRNAs showed significant regulation. Hence, we conclude that the modulation of Ago2 expression by Trim71 is not required to regulate miRNA expression in mES cells. However, this does not exclude a possible role of Ago2 in Trim71- or lin-41-dependent processes in other cell types and systems. Let-7 inhibition by lin-41 was shown to be dependent on Ago in the worm *C. elegans* to ensure neuronal plasticity in early development^73^. Interestingly, up-regulation of differentiation-promoting miRNAs in mES cells alone is not sufficient to induce differentiation as long as there is a steady expression of mES cell type miRNAs and pluripotency associated transcription factors in the cell^74^.

Recently, it has been demonstrated both on the cell population- and the single cell-level that mES cells exist at distinct sub-states also termed ground, transition and primed states^31^,^36^,^40^,^75^,^76^,^77^. External stimuli, blockade of Erk and GSK3, or disturbing the miRNA network, e.g. by loss of Dgcr8, led to the identification of a dynamic interconversion between these distinct states. More important, these findings also suggested that e.g. mES cells at the primed state are more primed towards differentiation and that the transition between stemness and differentiation is not necessarily a linear process. Our observations in the Trim71^−/−^ mES cells add another facet to these regulatory networks between stemness and differentiation. By uncoupling the mES-associated transcriptional network, which is intact in Trim71^−/−^ mES cells, from early steps required for neural development, we now postulate that the loss of the mES cell transcription factor and miRNA network is not necessarily an absolute requirement for lowering the threshold towards the earliest steps of differentiation. In fact, a recent model utilizing transient Nanog downregulation coupled with single-cell transcriptional profiling and mathematical modeling clearly revealed that early differentiation steps are stochastic and reversible in ES cells^31^. The authors elegantly demonstrated that a transient pluripotency state seems to be characteristic for a movement from a naive ES cell state to a primed state in which early differentiation programs and pluripotency circuitries are active in parallel. Our findings in Trim71^−/−^ mES cells suggests now that not only the transient loss of particular stemness factors, e.g. Nanog, but also the loss of the RBP Trim71 can induce differentiation priming in the presence of pluripotency programs.

## Methods

### Generation of a Trim71 targeted mouse line

All animal experiments were conducted in a licensed animal facility in accordance with the German law on the protection of experimental animals and were approved by local authorities of the state of Nordrhein-Westfalen (Landesamt für Natur, Umwelt und Verbraucherschutz NRW). The approval number is 87-51.04.2011.A063.

A *Mlin41/Trim71* targeted mouse line, named C57BL/6-Trim71^tm1695Arte^, was generated by Taconic Artemis (Cologne, Germany). For this purpose, 10–15 targeted C57BL/6N.tac ES cells were injected into Balb/c blastocysts (E3.5). After recovery, 8 blastocysts were transferred to each uterine horn of 2.5 days *post coitum* pseudopregnant NMRI foster females. Chimeric offspring showing germ line transmission were backcrossed into the C57BL/6J genetic background. For the generation of mice with an ubiquitous Cre-loxP recombined *Trim71* locus, males homozygous for the conditional *Trim71* allele were mated to females heterozygous for a *PGK-Cre* transgene where the Cre recombinase cassette is under the control of an ubiquitous and permanent active phosphoglyceratekinase (*PGK*) promoter^46^. In order to obtain animals with an inducible Trim71 locus, mice homozygous for the conditional Trim71 allele were crossed with mates homozygous for a Rosa26-CreER^T2^ transgene which harbours a Cre recombinase - estrogen receptor - fusion protein within the ubiquitously transcribed non-proteinogenic *Rosa26* locus on chromosome 1.

### Genotyping

For PCR genotyping, genomic DNA from tail biopsies of mice or from yolk sacs of embryos was extracted by boiling the tissue in an appropriate volume of 50 mM NaOH for 20 minutes and subsequent pH neutralization of the lysate by addition of a 1/4 volume of 1 M Tris-Cl, pH 8.0. For PCR the following primers were used: F1 5’-GAAAGGAGGCTAGCCAAAGG-3’, R1 5’-ATGCTGTACGGTAGGAGTCTTCC-3’, R2 5’-CACACAAAAAACCAACACACAG-3’. All PCR fragments were verified by sequencing.

### Plasmid constructs

The full-length human TRIM71 clone was obtained from F. Slack (Schulman *et al*., 2008) and the CDS was PCR amplified using the following primer sequences: 5’-GCG GGG ACG CGT ATG GCT TCG TTC CCC GAG ACC-3’ (contains MluI site) 5’-GCG GGG GCG GCC GCT TAA GAA GAC GAG GAT TCG ATT-3’ (contains NotI site and stop). The generation of the TRIM71 point mutant to substitute Cys 12 for Leu and Cys 15 for Ala was described elsewhere^72^.

For the generation of the TRIM71 RBCC variant the following reverse primer was used: 5’- TCC ACA TCT ACC GGG ATG AGC AGC GGG GCC TTT G -3’ (contains NotI site and stop). For amplification of the human TRIM32 cDNA primers with the sequences 5’-GCG GGG ACG CGT ATG GCT GCA GCA GCA GCT TC-3’ and 5’-GCG GGG GCG GCC GC CTA TGG GGT GGA ATA TCT TCT CAG ATG G-3’ were used. All constructs were cloned in a modified version of a pN1-eGFP or pRK5-flag vector, respectively, allowing MluI and NotI digestion.

### Histochemistry and *in situ* hybridization

For histological analysis, embryos within the decidua were embedded into paraffin, sectioned coronally at 8 μm thickness, dewaxed, and stained with hematoxylin and eosin. Whole mount *in situ* hybridizations using digoxigenin-labeled probes were performed as described previously^78^. For the synthesis of a riboprobe directed against Trim71 we cloned a fragment of the mRNA referring to nt 1999-2970 (NM_001042503.2) into the pGEM-T vector (Promega).

### Derivation of Trim71 conditional mES cells

For the derivation of conditional mES cell lines mice homozygous for the floxed Trim71 allele and the Rosa26-CreER^T2^ allele were used. Females were superovulated and early blastocysts were taken into culture and the dissociated inner cell mass was further expanded. For the induction of the Cre recombinase 250 nM 4-hydroxytamoxifen (Sigma) or DMSO as a vehicle control were added to the medium for 48 hours. Cells were further cultured for another 5 days before performing experiments. The mutation rate was determined using genotyping PCR for Trim71.

### Stem cell culture and differentiation procedures

Embryonic stem cells were cultured on gelatin coated culture dishes in MEF conditioned ES cell medium (Knockout DMEM (Invitrogen) supplemented with 15% FCS (Hyclone), Non-essential amino acids (PAA), Glutamaxx (Gibco), Penicillin-Streptomycin, beta-mercaptoethanol, and LIF (supernatant from L929 cells). Prior to differentiation experiments, the ES cell medium was supplemented with the compounds CHIR99021 (3 μM) and PD0325901 (1 μM) (Stemgent) in order to suppress background differentiation^36^. For EB differentiation, 5 × 10^6^ cells/ml were cultured in ES cell medium devoid of LIF and beta-mercaptoethanol in non-adhesive culture dishes. In order to investigate early neural differentiation, cells were cultured as EBs for 4 days and then dissociated using Trypsin. Cells were plated on PO/LN coated dishes and further cultured for four days in the presence of 100 nM retinoic acid. Medium was changed every other day.

For monolayer differentiation 1 × 10^4^ cells/cm^2^ were plated on gelatin coated plastic. Next day, mES cell medium was replaced with N2B27 medium (DMEM/F-12 supplemented with 1x N2 supplements, Neurobasal medium plus 1x B27 supplements, 1 mM L-glutamine, 1 mM nonessential amino acids, and 50 μg/ml BSA (fraction V)). Medium was changed daily.

### Generation of fetal radial glia-like neural stem cells

The generation and characterization of defined radial-glia-like neural stem cells from fetal mouse brains was described earlier^79^. Briefly, fetal cortical tissue (E14.5) was isolated and neural stem cells were expanded as floating neurospheres in NS-A media (Euromed-N media supplemented with N2 and containing 10 ng/ml EGF and 10 ng/ml FGF2). After 4-7 days, floating neurospheres were harvested and plated on PLO/LN-coated plastic dishes and further grown as an adherent culture in NS-A medium. Growth factors were added daily and the medium was changed every other day. For passaging, cells were trypsinized and replated on PLO/LN -coated dishes.

### Luciferase reporter assays

For the 3’UTR activity assays the psiCHECK^TM^-2 vector system from Promega was used inserting the 3’UTR sequences of murine Tcf15 (NM_009328.2), Plxnb2 (NM_001159521.2), Foxj1 (NM_008240.3), Inhbb (NM_008381.3), Mras (NM_008624.3), Nanos3 (NM_194059.2), Obscn (NM_199152.3), Prom1 (NM_008935.2) and Trim54 (NM_021447.2) using the restriction sites for XhoI and NotI. Transfection was carried out using Xfect mES reagent from ClonTech in a 24-well plate scale using 500 ng DNA per well. Luciferase activities were measured 24 hours after transfection using the Dual Luciferase Assay System (Promega) and the MicroLumatPlus LB96V (Berthold Technologies) according to the manufacturer’s instructions. Results are mean ratios of Renilla to Firefly activity normalized to Trim71^fl/fl^ levels.

### Flow cytometry and microscopy

For SSEA-1 surface staining 3 × 10^5^ cells per sample were incubated with α-SSEA-1 Alexa Fluor 647 (clone MC-480) for 20 min at 4 °C. For co-staining, the α-SSEA-1 antibody was combined with α-Plexin B2-PE (clone 3E7), α-CD133-PE (clone 13A4) or the respective isotype control antibody.

For confocal microscopy eGFP-Trim71 transfected JKT-1 cells were seeded on glass cover slips coated with Poly-L-lysine, fixed with 4% PFA, permeabilized with 0,2% Triton X-100/PBS. The cells were first incubated with anti-Ago2 antibody (Cell Signaling) 1:200 diluted and incubated for 1 hour at RT, and subsequently treated with an Alexa Fluor-647-coupled anti-rabbit secondary antibody (Jackson ImmunoResearch) and DAPI for another hour. Images were taken using an Olympus Fluoview 1000 confocal microscope equipped with a Plan Apochromat 60×, NA 1.4 oil immersion objective (Olympus) and DIC.

### Western blot analysis

Cell samples were lysed in a buffer containing 150 mM NaCl, 10 mM HEPES, 2 mM MgCl_2_, 10 mM KCl, 0,5 mM EDTA and 0.5% Triton X-100. Protease inhibitors were added freshly. The Protein concentration was measured using the Pierce BCA Protein Assay Kit (Thermo Fisher) and equal amounts of protein were separated by electrophoresis on SDS/PAGE and blotted onto cellulose membranes (BioTrace NT, Pall). Membranes were blocked with 3% BSA in TBST and then incubated with the primary antibody over night at 4 °C (*i.e*. sheep anti-Trim71 (R&D Systems) rabbit anti-Ago2 (Cell Signaling) rabbit anti-TuJ1 (Cell Signaling), rabbit anti-Sox2 (Cell Signaling), mouse anti-Tubulin (Sigma).

After washing with TBST, followed by incubation with HRP-conjugated secondary antibodies (Jackson ImmunoResearch), bands were detected by chemiluminescence (Thermo Scientific).

### RNA isolation

For RNA isolation 5 × 10^6^–2 × 10^7^ mES cells were harvested, subsequently lysed in TRIzol (Invitrogen), and total RNA was extracted according to the manufactures’ protocol. The precipitated RNA was solved in RNAse free water. The quality of the RNA was assessed by measuring the ratio of absorbance at 260 nm and 280 nm using a Nanodrop 2000 Spectrometer (Thermo Scientific) as well as by visualization the integrity of the 28S and 18S band on an agarose gel.

### RT-qPCR

RNA was extracted using the TRIzol method and following DNaseI treatment cDNA was synthesized using the High Capacity cDNA Reverse Transcription Kit (Applied Biosystems) according to the manufacturer’s instructions. For semi-quantitaive PCR 10 ng of cDNA were used to run 30 amplification cycles with primers for Trim71 (5’-GTGGAAGGTGGAGAAGATCCG-3’ and 5’-GTCAGGGGGCGTGAACATAA-3’) or Hprt (5’-GCTGGTGAAAAGGACCTCT-3’ and 5‘-CACAGGACTAGAACACCTGA-3‘). For quantitative PCR gene specific probes from Applied Biosystems were used in combination with the PeqGold Hot Start Mix (Peqlab). Gapdh mRNA was used as housekeeping gene. All graphs represent mean values +SEM or +SD from at least three independent experiments.

### Generation of cDNA libraries

Total RNA was converted into libraries of double stranded cDNA molecules as a template for high throughput sequencing following the manufacturer’s recommendations using the Illumina TruSeq RNA Sample Preparation Kit v2. Shortly, mRNA was purified from 100 ng of total RNA using poly-T oligo-attached magnetic beads. Fragmentation was carried out using divalent cations under elevated temperature in Illumina proprietary fragmentation buffer. First strand cDNA was synthesized using random oligonucleotides and SuperScript II. Second strand cDNA synthesis was subsequently performed using DNA Polymerase I and RNase H. Remaining overhangs were converted into blunt ends via exonuclease/polymerase activities and enzymes were removed. After adenylation of 3′ ends of DNA fragments, Illumina PE adapter oligonucleotides were ligated to prepare for hybridization. DNA fragments with ligated adapter molecules were selectively enriched using Illumina PCR primer PE1.0 and PE2.0 in a 15 cycle PCR reaction. Size-selection and purification of cDNA fragments with preferentially 200 bp in length was performed using SPRIBeads (Beckman-Coulter). The size-distribution of cDNA libraries was measured using the Agilent high sensitivity DNA assay on a Bioanalyzer 2100 system (Agilent). cDNA libraries were quantified using KAPA Library Quantification Kits (Kapa Biosystems). After cluster generation on a cBot, a 2 × 100 bp paired-end run was performed on a HiSeq1500.

### RNA-seq preprocessing

After base calling and de-multiplexing using CASAVA version 1.8 the 100 bp paired-end reads were aligned to the murine reference genome mm10 from UCSC by TopHat2 version v2.0.11 and Bowtie2 version 2.2.1 using the default parameters. This annotation included 32.600 unique transcript entries with genomic coordinates. After mapping of the reads to the genome, we imported the data into Partek Genomics Suite V6.6 (PGS) to calculate the number of reads mapped to each transcript against the RefSeq mm10 annotation download on April 2014. These raw read counts were used as input to DESeq2 for calculation of normalized signal for each transcript^80^ using the default parameters. After DESeq2 normalization, the normalized read counts were imported back into PGS and floored by setting all read counts to at least a read count of 1 after the batch-correction. Subsequent to flooring, all transcripts having a maximum over all group means lower than 10 were removed. After dismissing the low expressed transcripts the data comprised of 13.558 transcripts. RNA-seq data can be accessed under GSE62398.

### miRNA-seq

Sequencing of miRNAs was done according to the manufacturer’s recommendations. In brief, 5 × 10^6^ cells were harvested and total RNA including small RNAs was isolated. Small RNA libraries were generated from 500 ng total RNA with the NEBNext Multiplex Small RNA Library Prep Set (New England Biolabs). After successful ligation of the 3’ adaptor to RNA molecules, the reverse transcription primer was added before ligation of the 5’ adapter. Next, RNA was reverse-transcribed using ProtoScript II Reverse Transcriptase. cDNA was amplified by 15 PCR cycles with high-fidelity LongAmp Taq 2X Master Mix and the respective index primer. The indexed libraries with the size of miRNAs plus ligated adapters were quantified on the Roche LightCycler 480 II qPCR instrument using the KAPA Library Quantification Kit in triplicates, according to the manufacture’s protocol (Kapa Biosystems, Woburn, MA, USA). After pooling of equimolar ratios of indexed libraries these were purified on a pre-cast 6% Tris/Borate/EDTA polyacrylamide electrophoresis gel (Invitrogen). Generation of clonal clusters from single molecules of the cDNA library was done with the TruSeq Cluster Kit (Illumina) on a CBot station. Sequencing by synthesis was performed by using the TruSeq SBS Kit on a HiSeq 1500 system (Illumina). Sequencing reads were retrieved as FASTQ files.

### miRNA-seq preprocessing

After base calling, de-multiplexing and trimming using CASAVA version 1.8 and FLICKR the 50 bp reads were aligned to the murine reference genome mm10 from mirBase v20^81^ by Bowtie2 version 2.2.1 using the default parameters. SAMtools version 0.1.18^82^ was then used to convert the SAM files into smaller BAM files. The annotation included 24.521 unique transcript entries with genomic coordinates. After mapping of the reads to the genome, we imported them into Partek Genomics Suite V6.6 (PGS). To calculate the number of reads mapped to each transcript we generated a custom annotation file based on the BAM files using the integrated function in PGS. The normalization and post-processing of the data was performed the same way as described for RNA-seq processing. After dismissing the low expressed transcripts the data contained 590 miRNA transcripts. miRNA-seq data can be accessed under GSE62509.

### Statistical and descriptive bioinformatics of transcriptome data

A one-way ANalysis Of VAriance (ANOVA) model was performed to calculate the 1.000 most variable and the differentially expressed genes (transcripts) between Trim71^fl/fl^ and Trim^−/−^ mES cells using PGS. Differentially expressed genes were defined by a fold change (FC) > 1.5 or < −1.5 and a p-value < 0.05. To visualize the structure within the data we performed Principle Component Analysis (PCA) on all genes and hierarchical clustering (HC) on the 1.000 most variable genes, with default settings in PGS, based on p-values according to the expression values of the samples across the conditions. In order to show whether the loss of Trim71 can be verified on the transcript level, read counts were visualized by IGV^83^ mapping the aligned reads against the mouse genome (mm10).

To identify the differences and similarities between Trim71^fl/fl^ and Trim71^−/−^ mES cells the differentially expressed genes were visualized with SigmaPlot version 12.0 (Systat Software, San Jose, CA) as a ratio-ranked (log_2_) plot. The expression of known important ES transcription factors were visualized as a heatmap by Mayday^84^. To link differentially expressed genes to known biological functions we used the 166 most differentially upregulated and 216 most downregulated genes in Trim71^−/−^ compared to Trim71^fl/fl^ mES cells in order to generate and visualize a network based on GO-enrichment analysis (GOEA) by using BiNGO^85^, EnrichmentMap^86^, and Word Cloud^87^ in Cytoscape^88^.

### Descriptive bioinformatics of miRNome

A one-way ANalysis Of VAriance (ANOVA) model was performed to calculate the 100 most variable and the differentially expressed miRNAs between Trim71^fl/fl^ and Trim^−/−^ mES cells using PGS. Among the 590 miRNA differentially expressed miRNAs were defined by a fold change (FC) > 1.5 or < −1.5 and a p-value < 0.05. To visualize the structure within the data we performed PCA on all miRNAs and hierarchical clustering (HC) on the 100 most variable miRNAs, with default settings in PGS, based on p-values according to the expression values of the samples across the conditions. Mean ratios of differentially expressed miRNAs between Trim71^fl/fl^ and Trim^−/−^ mES cells were plotted against the maximum abundance. Log2-transformed read counts of differentially expressed miRNAs were visualized as a heatmap using MayDay. For tissue-related classification, we utilized miRNA-seq data by Chiang *et al*.^62^ who measured the relative miRNA expression in various mouse tissues and developmental stages (ovary, testis, brain, newborn, ESC, E7.5, E9.5, and E12.5 embryos). MiRNAs were further categorized as proposed by Jouneau *et. al*.^65^ by showing mainly expression (>50% of reads) in one of the following categories: mES cells, gonads (ovary and testis), brain, other somatic tissue (newborn, E12.5, and E9.5). MiRNas not fulfilling these criteria e.g. miRNAs with similar expression in all categories, were classified as unspecified.

### Overlay of Trim71-associated transcriptome data on the ES cell network

In order to evaluate the influence of Trim71 gene deficiency on stemness we generated a mES cells specific gene network based on reverse network engineering and co-regulation analysis (schema in Fig. 4a). Information-theoretic methods such as the TINGe (Tool for Inferring Network of Genes) algorithm^52^ have been introduced to determine most highly connected genes within a dataset. To determine the most highly connected genes reflecting the core mES cell network, transcriptional interactions between genes were determined by TINGe. For this analysis we used 165 arrays of a previously described study (GSE19836 and GSE32015)^51^. The 9.027 unique present genes were taken into calculation of mutual information (a measure of the mutual dependence of the two genes as random variables) with p-value less than 1e-7 and a mutual information cutoff of 0,4 (Suppl. Fig. 2). The threshold of the data processing inequality (DPI) from information theory used by TINGe was set to 0.1 and used to detect and discard indirect interactions that are unlikely to be mediated by an actual physical interaction. The functional relationship between the numbers of nodes and their degree of interactions was estimated by power law regression (Suppl. Fig. 2). The R package poweRlaw was used to validate whether the TINGe network is following the power law distribution. The power law regression in the relationship between the number of nodes (number of genes ranging from 645 to 1) and their degree (number of interactions ranging from 1 to 105) suggests a scale-free network structure, i.e. the network is unevenly populated with highly connected nodes and less dense nodes. The R squared value was 0.763 and the heavy-tailed distribution, indicating high correlation and a strong linear relationship between degree of connectivity and corresponding number of genes. The network was visualized in a force-directed layout in Cytoscape (Fig. 4c (ES filtered) and Suppl. Figure 2 (10% most highly connected genes)). Subsequently we mapped the fold change differences in expression between Trim71^fl/fl^ and Trim71^−/−^ mES cells onto the TINGe network.

To validate the structure in the data we used a co-regulation analysis based on Pearson’s correlation coefficients by using BioLayout Express3D (Pearson correlation cutoff of 0.55)^58^. The network was visualized by Cytoscape (Fig. 4d) and was filtered by a set of 454 unique genes that were previously described by de Cegli *et. al*. to be ES cell specific^51^. Subsequently we mapped the fold change differences in expression between Trim71^fl/fl^ and Trim71^−/−^ mES cells onto the co-regulation network.

### Overlay of Trim71-regulated genes on time kinetics data of neuronal cell development

In order to evaluate the developmental status of Trim71^−/−^ mES cells, we performed topological mapping based on a data set (PRJNA185305) that comprises Illumina HiSeq 2000 data of 31 samples of different stages of neuronal development^65^. Following normalization using DESeq with default parameters the normalized read counts were imported into Partek Genomics Suite (PGS), a batch correction was performed and all read counts were set to at least a read count of 1. After dismissing all transcripts having a maximum over all group means lower than 10 the data were comprised of 16.878 present transcripts. A one-way ANOVA (|FC| > 2, FDR-corrected p-value < 0.05) was performed and was used to determine differential expressed genes between mES cells and the later stages in neuronal development (NESC, RG, DS I/II neurons and MDS IV/V neurons). The 200 most upregulated genes at each time point were used as specific marker genes for the respective stage, whereas the 200 most down regulated genes among all time points were used as specific marker genes for mES cells. This resulted in a total number of 1.029 genes linked to different states in neuronal development. Furthermore, we included differentially expressed genes between Trim71^−/−^ and Trim71^fl/fl^ mES cells (|FC| > 2, p-Value < 0.05) and genes associated with neuronal development in Fig. 3f into our analysis. To generate a stage-specific topological structure based on these genes, we performed co-regulation analysis (CRA) based on Pearson’s correlation coefficients by using BioLayout Express3D (Pearson correlation cutoff of 0.90)^66^. The co-regulation network was visualized by Cytoscape and colored by the top 200 stage specific marker genes of each developmental stage, which allows the definition of topological subnetworks by accumulation of genes specific for a particular developmental state.

In order to evaluate changes in developmental state caused by Trim71 deficiency we determined differentially expressed genes between Trim71^−/−^ and Trim71^fl/fl^ mES cells by a two-way ANOVA model (|FC| > 1.4, unadj. p-value < 0.05). Next, we mapped the differentially expressed genes of Trim71^−/−^ mES cells onto the co-regulation network describing neuronal development. Furthermore, the genes linked to later differentiation states were analyzed by GOEA using BiNGO, EnrichmentMap, and Word Cloud in Cytoscape.

As a complementary strategy we used the top differentially expressed genes between Trim71^−/−^ and Trim71^fl/fl^ mES cells that mapped to the co-regulation network, determined the expression values of these genes in the neuronal development dataset (PRJNA185305), ordered the genes according to their highest expression along the developmental path towards neurons and visualized their expression values during neuronal development as a heatmap.

### GOEA of ESCs differentiated into either endodermal, ectodermal or mesodermal germ layers

To determine changes in differentiation initiation in endodermal, ectodermal or mesodermal germ layers two existing datasets (GSE11523: primitive endoderm, primitive ectoderm/neural ectoderm and GSE58300: cardiomyocte-specific differentiation from ES cells as example for mesodermal cell development) were compiled and GOEA was applied by using the GO enrichment tool of PGS. Data were imported into PGS and quantile normalized. Genes were only kept for further analysis if their mean expression values were higher than 7.5 (for GSE11523) or 5.5 (for GSE58300), which resulted in 13572 (endoderm), 13224 (ectoderm) and 13568 (mesoderm) present genes. A one-way ANOVA (FC > 3, FDR-corrected p-value < 0.05) was performed to determine differentially expressed genes between ES cells and the final three differentiation stages of each germ layer. Next, genes were identified which were both upregulated during germ-layer differentiation and differentially expressed (|FC| > 1.4 FDR-corrected p-value < 0.05) between Trim71^−/−^ and Trim71^fl/fl^ mES cells. Only genes which fulfilled this criterion were used for further analysis. GOEA was performed for each germ layer separately and the most highly enriched GO terms (top 25) downstream of the GO term ‘development’ within the GO database tree ‘biological process’ were visualized as a hierarchical tree structure and as a heatmap.

## Additional Information

**How to cite this article**: Mitschka, S. *et al*. Co-existence of intact stemness and priming of neural differentiation programs in mES cells lacking Trim71. *Sci. Rep*. **5**, 11126; doi: 10.1038/srep11126 (2015).

## Supplementary Material

Supplementary Information

Supplementary Table S1

Supplementary Table S2

Supplementary Table S3

Supplementary Table S4
