# Supplementary material for: Co-existence of intact stemness and priming of neural differentiation programs in mES cells lacking Trim71
Source: Sci Rep. 2015 Jun 9;5:11126. doi: 10.1038/srep11126 (PMC4460895; doi:10.1038/srep11126)

Supplementary Information

Co-existence of intact stemness and priming of neural differentiation programs in mES cells lacking Trim71

Sibylle Mitschka[1,6], Thomas Ulas[2,6], Tobias Goller[1], Karin Schneider[1], Angela Egert[3], Jérôme Mertens[4], Oliver Brüstle[4], Hubert Schorle[3], Marc Beyer[2], Kathrin Klee[2], Jia Xue[2], Patrick Günther[2], Kevin Bassler [2], Joachim L. Schultze[2], and Waldemar Kolanus[1, 5]

[1] Molecular Immunology and Cell Biology, Life and Medical Sciences Institute, University of Bonn, Germany

[2] Genomics and Immunoregulation, LIMES-Institute, University of Bonn, Bonn, Germany

[3] Department of Developmental Pathology, Institute of Pathology, University of Bonn Medical School, Bonn, Germany

[4] Institute for Reconstructive Neurobiology, Life and Brain Center, University of Bonn and Hertie Foundation, Bonn, Germany

[5] Contact:
email: wkolanus@uni-bonn.de
Phone: +49-228-62790
Fax: +49-228-62647

[6] The contribution of the first two authors should be considererd equal

Running title: Regulation of neural differentiation by Trim71 in mES cells

Suppl. Figure 1: A new Trim71 knock-out allele leads to embryonic lethality in mice

(a) Trim71 locus in wild-type, conditional and knock-out allele **(b)** Genotype statistics of analyzed litters from heterozygous intercrosses at different developmental stages showing that homozygous Trim71 mutation leads to death of embryos before birth. In contrast, heterozygous offspring appears to be normal. Numbers in brackets indicate average litter size for decidua and percentage of total embryos for genotype categories. **(c)** Lateral (left panel) and anterior (right panel) view of $Trim71^{+/+}$ and $Trim71^{-/-}$ embryos at stage E10.5 (18x magnification) shows growth retardation and neural tube closure defect of Trim71 deficient embryos.

Suppl. Figure 2: Reverse engineering network of mES cells

(a) Schematic representation of the experimental setup. **(b)** Degree node distribution of the network generated using TINGe (mutual information cut-off of 0.4). The number of genes with the same number of interactions (from 1 to 105) fits to a power law (dash line) in logarithmic range. This indicates that the network is scale-free. **(c)** TINGe predicted network showing 10% most highly connected genes visualized using Cytoscape. All gene names are shown.

Suppl. Figure 3: GOEA of ES cells differentiated into either endodermal, ectodermal or mesodermal cells

(a) Schema describing the workflow for Suppl. Figure 4. Genes, which were both upregulated during germ-layer differentiation and differentially expressed between $Trim71^{-/-}$ and $Trim71^{fl/fl}$ mES cells, were used for GOEA. **(b)** Venn diagrams showing intersection of genes which were differentially expressed (either upregulated or downregulated) during germ layer differentiation and genes whose expression were affected due to Trim71 knockout in mES cells. **(c)** Schema showing the hierarchical organization of GO terms. **(d)** For each germ-layer the top 25 highly enriched GO terms were determined and those which were related to development were visualized in a hierarchical tree

structure. Node border color and thickness represent corresponding E-score, whereas light red filled nodes show GO terms which were also found to be enriched in data set PRJNA185305. Additionally, GO terms related to development were also used for generating a heatmap **(e)**, in which the respective E-score for each germ layer is visualized.

Suppl. Figure 4: Generation of a co-regulation network of neuronal cell development.

(a) Schematic representation of the experimental setup; ESC: embryonic stem cell, NESC: neuroepithelial stem cell, RG: radial glia, DS: developmental stage, MDS: mature developmental stage. The co-regulation network was generated based on Pearson's correlation coefficients by using BioLayout Express3D (Pearson correlation cutoff of 0.9) and visualized in Cytoscape. **(b)** For each differentiation state the 200 most differentially expressed genes were mapped onto the network to better visualize the overall topology of neuronal development within the network. The color coding is based on the log2 transformed mean expression values for the marker genes specific for each developmental stage.

Suppl. Figure 5: Additional Information on candidate genes tested on Trim71 mediated expression regulation

(a) List of selected candidate genes which were unchanged, upregulated or downregulated in Trim71$^{-/-}$ mES cells. [1] Data were obtained from the respective reference transcripts deposited on NCBI. [2] Annotations according to TargetScan Release 6.2. [3] This 3'UTR was annotated differentially in NCBI database and TargetScan 6.2. Besides, there were no references for miRNA binding sites.
(b) Principle of the luciferase reporter assay used to analyze Trim71 dependent posttranscriptional expression regulation.

Suppl. Figure 6: Trim71 interacts with Ago2, but does not affect Ago2 protein stability

(a) eGFP-tagged TRIM71 was overexpressed in the human EC cell line JKT-1 and 24 hours post transfection cells were fixed for staining of endogenously expressed AGO2. TRIM71 co-localizes with AGO2 in cytoplasmic processing bodies (arrows). **(b)** Co-IP in Hek293T cells after overexpression of different TRIM71 constructs shows that wild-type TRIM71 interacts with AGO2. In contrast, the double point mutant (C12L/C15A) in the RING domain of TRIM71, a mutant consisting only of the RBCC motif and the structurally related TRIM-NHL protein TRIM32 show much weaker interaction. **(c)** Trim71 deficient mES cells have an unaltered Ago2 protein amount and mRNA expression **(d)** in comparison to control cells.

Suppl. Figure 7: Trim71 deficiency enhances the expression of neuroectodermal marker genes during N2B27 differentiation

Trim71$^{fl/fl}$ and Trim71$^{-/-}$ mES cells were differentiated for 7 days in N2B27 medium and analyzed for Sox1 **(a)** and Pax6 **(b)** expression at different time points by RT-qPCR. Data were normalized to marker levels in Trim71fl/fl cells in undifferentiated state (mES) and represent mean +SEM of 4 independent experiments.

Suppl. Figure 8: Radial glia like stem cells do not express Trim71

(a) Primary neural stem (NS) cells were isolated from E14.5 Trim71$^{fl/fl}$ /Cre-ER^{T2} animals and cells were analyzed for their miRNA expression. In comparison to mES cells the expression of ESCC miRNAs miR-294 and miR-302a is strongly downregulated whereas let-7a is induced in NS cells. **(b)** Genotyping PCR showing the conversion of the floxed Trim71 allele to the deleted allele in neural stem cells after 48 hour treatment with 250 nM 4-OHT. DMSO treated cells and wild-type Trim71 cells with Rosa26-CreER^{T2} background served as a control. Upper band: floxed allele; middle band:

knock-out allele; lower band: wild-type allele. **(c)** No morphological changes were observed after 4-OHT treatment. Scale bar represents 100 µm. **(d)** Radial Glia-like neural stem cells do not express the Trim71 protein any longer, therefore the deletion has no effect on the protein expression of typical neural stem cell markers such as Sox2, TuJ**. (e)** RT-qPCR confirms massive downregulation of Trim71 mRNA in NS cells in comparison to mES cells. The same applies to Oct4 but not the NS cell marker Sox2.

Suppl. Figure 1

a

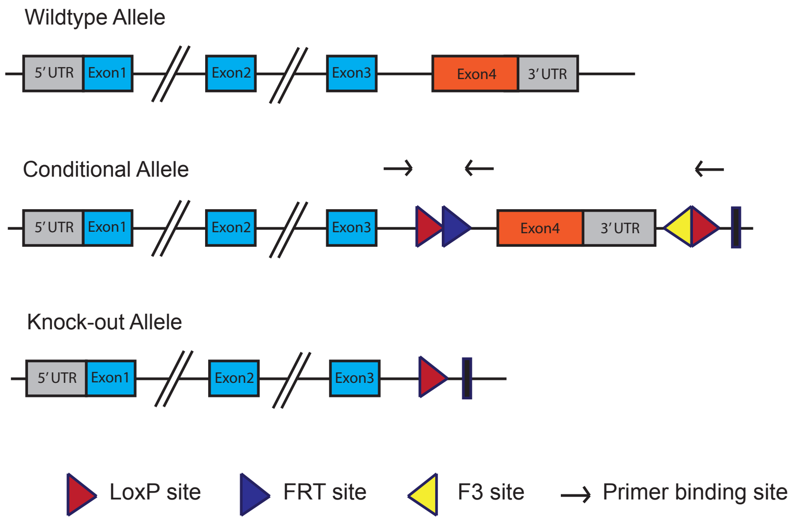

b

Stage	Litters	Decidua	+/+	+/-	-/-	resorbed
**E8.8**	30	218 (7.3)	61 (28.0%)	83 (38.1%)	61 (28.0%)	13 (6.0%)
**E9.5**	60	448 (7.5)	113 (25.2%)	193 (42.9%)	119 (26.6%)	24 (5.4%)
**E10.5**	14	108 (7.7)	21 (19.4%)	49 (45.4%)	29 (26.9%)	9 (8.3%)
**E11.5 -E14.5**	9	59 (6.6)	9 (15.3%)	30 (50.8%)	10 (16.9%)	10 (16.9%)
**adult**	22	80 (3.6)	32 (40.0%)	48 (60.0%)	0 (0%)	- -

c

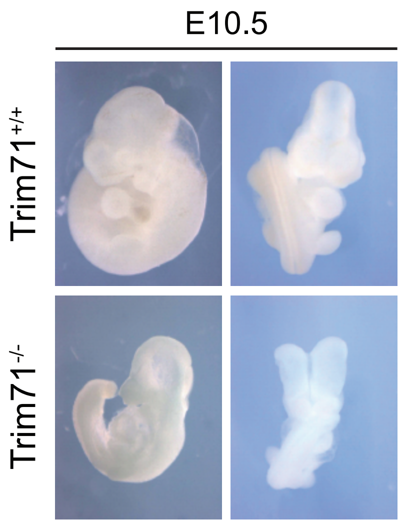

Suppl. Figure 2

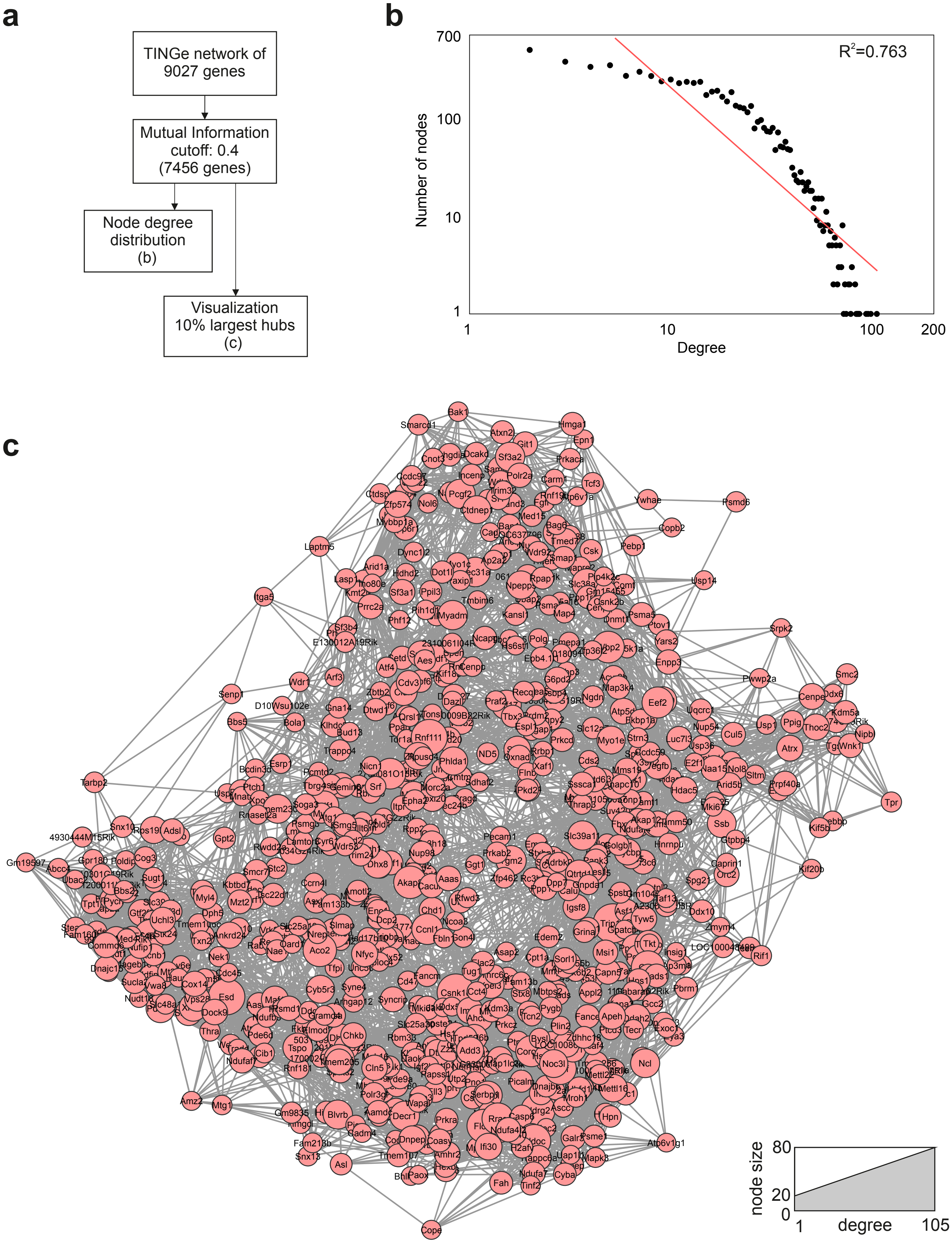

Suppl. Figure 3

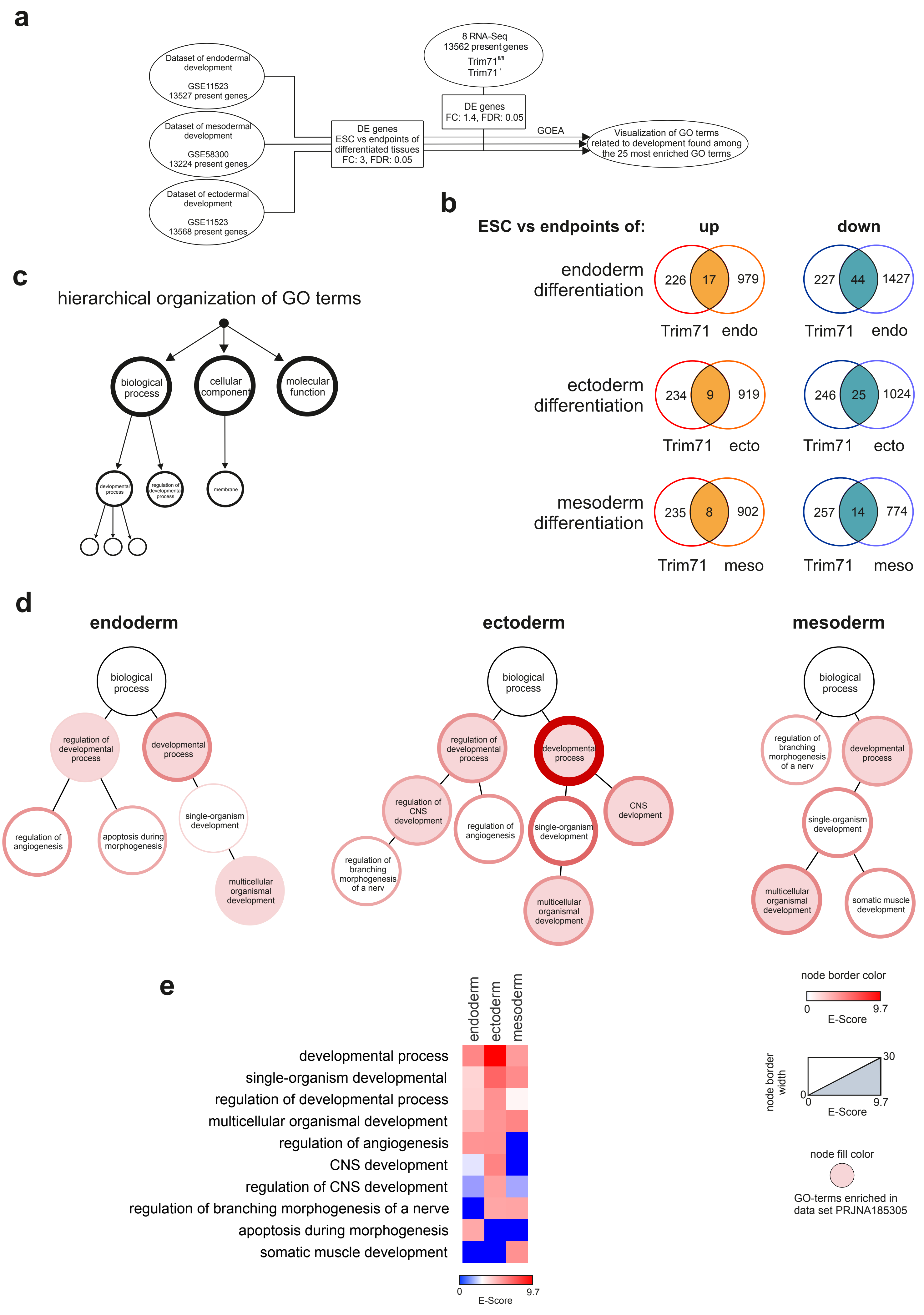

Suppl. Figure 4

a

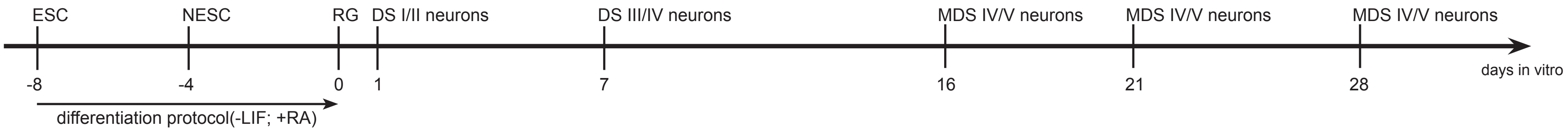

b

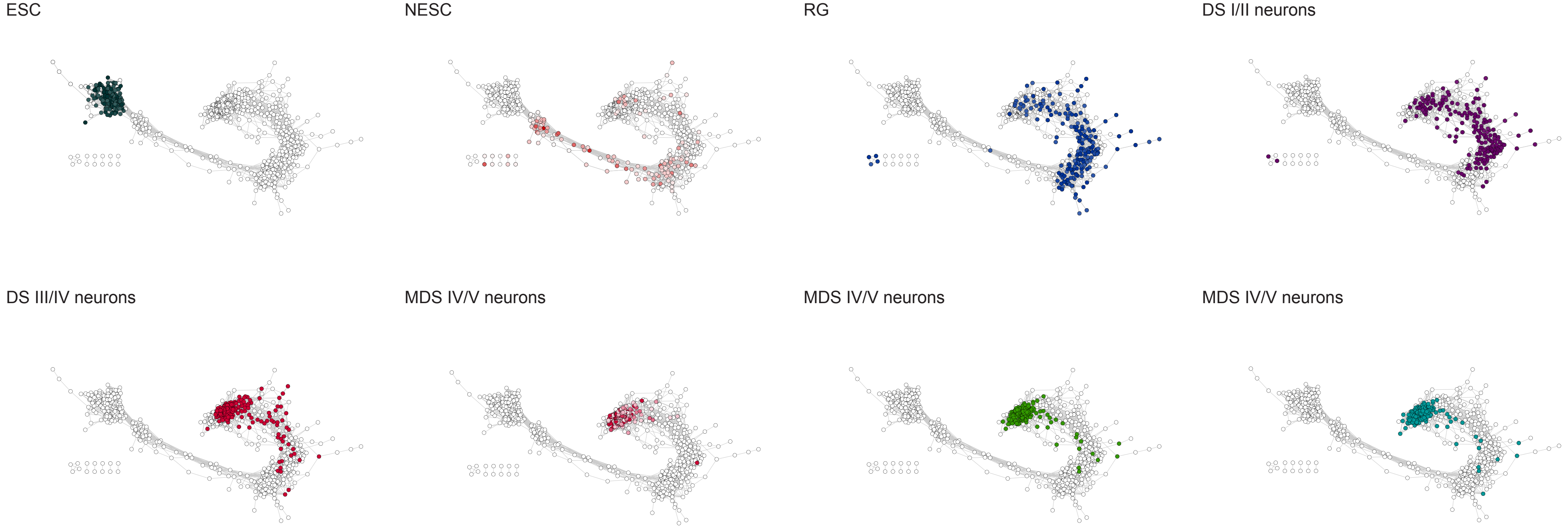

Suppl. Figure 5

a

Gene	Function	3'UTR[1]	Broadly conserved miRNA target sites[2]
Pou5f1	Transcription factor essential for early embryogenesis and embryonic stem cell pluripotency	264 bp	none
Tcf15	Early transcriptional regulator of (mesodermal) differentiation	344 bp	none
Plxnb2	Transmembrane receptor required for normal differentiation and migration of neuronal cells during brain corticogenesis and for normal embryonic brain development	754 bp	miR-124ab/506<br>miR-137ab<br>miR-138ab<br>miR-192/215
Foxj1	Transcription factor that is involved in the formation of motile cilia, implicated in establishment of left-right-asymmetry, lung development, postnatal neurogenesis	1027 bp	miR-141/200a
Inhbb	Protein subunit of inhibins and activins which act as both a growth/differentiation factor and a hormone, especially in the gonads	1857 bp	>5 sites
Mras	Membrane-anchored, intracellular signal transducer regulating various processes	3266 bp	>5 sites
Nanos3	RNA-binding protein regulating germ cell maintenance	215 bp	none
Obscn	Signaling and anchor protein involved in the organization of myofibrils in striated muscle	495 bp	let-7/98/4458/4500
Prom1	Pentaspan transmembrane glycoprotein expressed in adult stem cells and suppressing differentiation	1167 bp	miR30a-f/384<br>miR-203
Trim54	E3-ubiquitin ligase regulating titin kinase and microtubule-dependent signal pathways in striated muscles	149 bp	none [3]

b

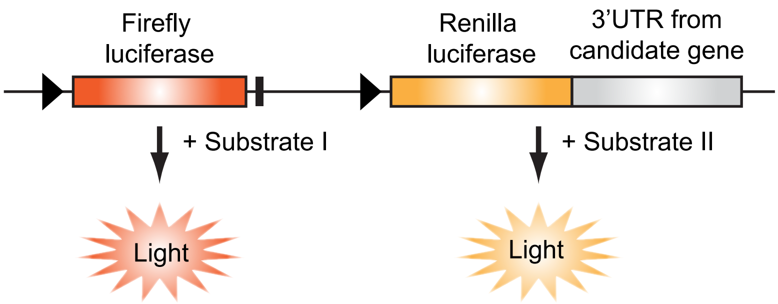

Suppl. Figure 6

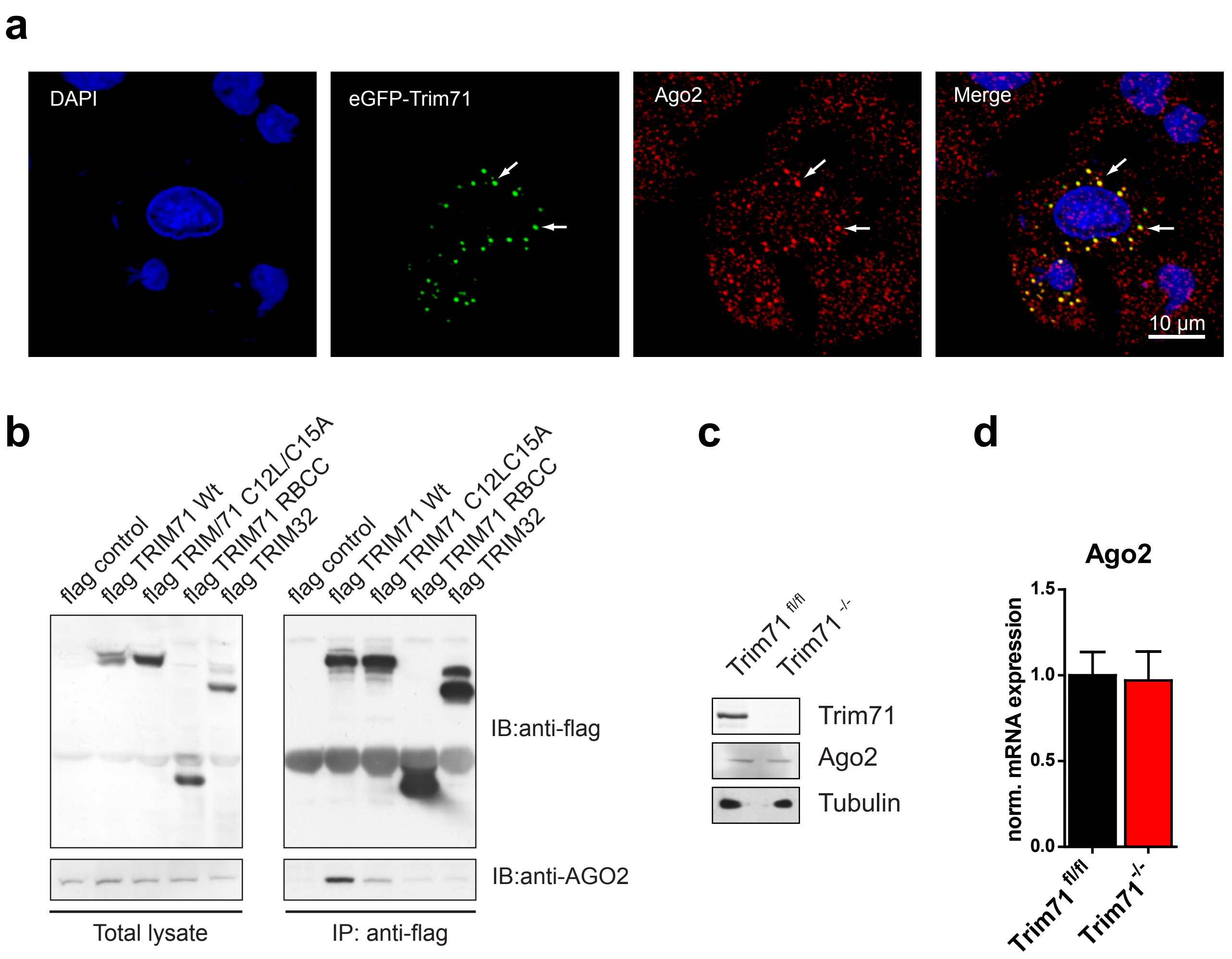

Suppl. Figure 7

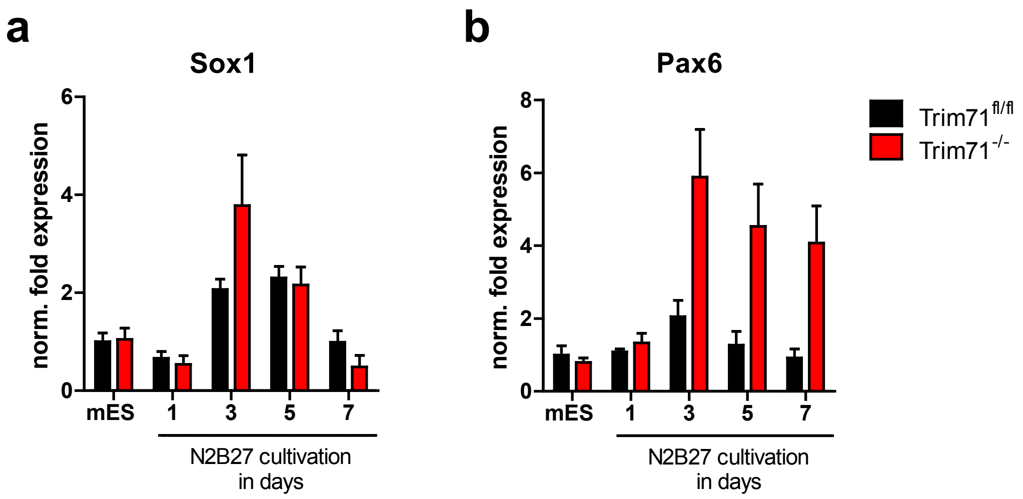

Suppl. Figure 8

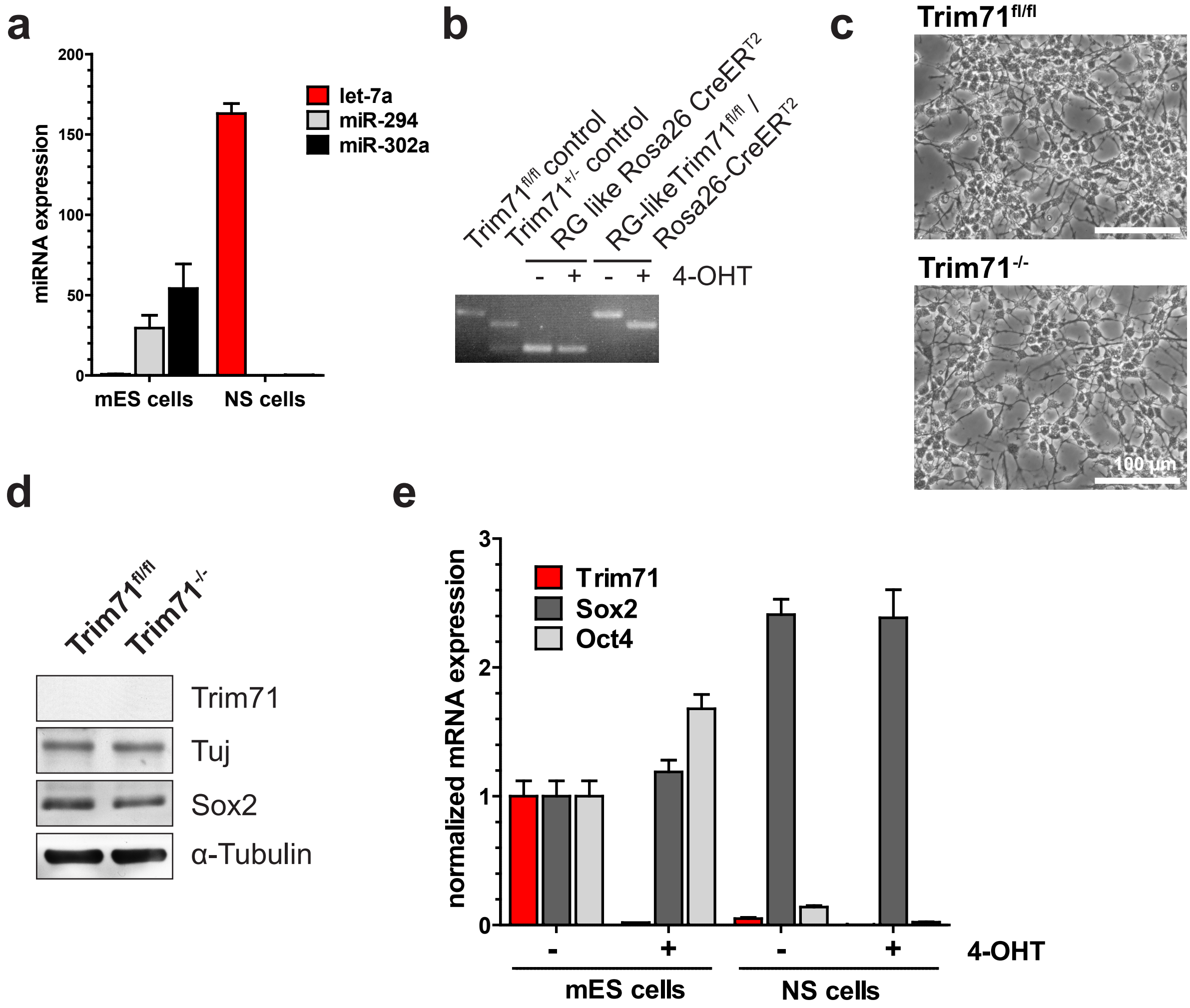

Supplement: Supplementary Information [file srep11126-s1.pdf]
